# Attenuation of blood pressure in spontaneously hypertensive rats by acupuncture was associated with reduction oxidative stress and improvement from endothelial dysfunction

**DOI:** 10.1186/s13020-016-0110-0

**Published:** 2016-08-30

**Authors:** Sin Bond Leung, Hongwei Zhang, Chi Wai Lau, Zhi-Xiu Lin

**Affiliations:** 1 School of Chinese Medicine, Faculty of Medicine, The Chinese University of Hong Kong, Shatin, N.T., Hong Kong, People’s Republic of China; 2 School of Biomedical Sciences, Faculty of Medicine, The Chinese University of Hong Kong, Shatin, N.T., Hong Kong, People’s Republic of China

**Keywords:** Acupuncture, Hypertension, Endothelial dysfunction, Spontaneously hypertensive rats

## Abstract

**Background:**

Hypertension can be treated effectively by acupuncture; however, the association between acupuncture and endothelial function remains unknown. This study aimed to investigate the effects of acupuncture on endothelial dysfunction and oxidative stress-related parameters in spontaneously hypertensive animals.

**Methods:**

Eighteen-week-old Wistar–Kyoto rats (WKYs) and spontaneously hypertensive rats (SHRs) were arbitrarily divided into four groups: WKY control (n = 8), SHR control (n = 8), SHR sham-acupuncture (n = 8) and SHR acupuncture (n = 8). The SHR acupuncture group had electroacupuncture for 6 consecutive weeks on acupoints ST36 and LR3. Blood pressure was monitored during the treatment period, and animals were euthanized at the 6th week. Aortas were harvested for determination of angiotensin II levels, NADPH oxidase activity and nitrate/nitrite levels. The level of reactive oxygen species (ROS) was determined by dihydroethidium (DHE) imaging, and functional studies were performed to assess vascular reactivity. Endothelial nitric oxide synthase was measured by Western blot assay.

**Results:**

Blood pressure at the end of treatment was significantly lower in the SHR acupuncture group (185.0 ± 5.6 mmHg) compared with the SHR sham-acupuncture and the SHR control groups (201.0 ± 5.4 and 197.4 ± 5.9 mmHg, respectively; *P* < 0.001). Serum angiotensin II level in the SHR control group was significantly higher than in the WKY control group (*P* < 0.001), while it was significantly attenuated by acupuncture treatment (*P* = 0.023). DHE staining showed that ROS level was reduced in the aortas (*P* = 0.0017) and carotid arteries (*P* = 0.039) of acupuncture-treated SHRs. Biochemical assays showed that acupuncture inhibited the NADPH oxidase activity (*P* = 0.022) and enhanced antioxidant capacity (*P* = 0.0039). In functional studies, endothelium-dependent relaxation of aortic rings (*P* = 0.018) and carotid arteries (*P* = 0.022) in response to acetylcholine was improved in the SHR acupuncture group. Aortas of SHRs receiving acupuncture also expressed an elevated level of eNOS (*P* > 0.001) and p-eNOS (*P* = 0.012) and a reduced nitrotyrosine level (*P* = 0.0012). The nitrate/nitrite level in aortic tissue was also attenuated after acupuncture (*P* = 0.0018).

**Conclusion:**

The effects of acupuncture in treating hypertension were associated with reduced oxidative stress, increased nitric oxide bioavailability and endothelial function in SHRs.

**Electronic supplementary material:**

The online version of this article (doi:10.1186/s13020-016-0110-0) contains supplementary material, which is available to authorized users.

## Background

Acupuncture is recognized by the World Health Organization (WHO) for the management of many diseases, including hypertension [1]. Previous studies have showed that acupuncture reduces hypertension through diverse mechanisms [2, 3]. Acupuncture intervention re-modulated the activity of the renin-angiotensin-aldosterone system, endothelium-derived vasoactive substances, and vascular function [4–6]. The effects of acupuncture on hypertension might be associated with regulation of NO [1]. It was reported that NO levels increased after acupuncture treatment [7–9]. Moreover, acupuncture exhibited strong antioxidative effects in the treatment of cerebral ischemia, myocardial injury, and Parkinson’s disease [10–14]. However, the effects of acupuncture on hypertension in relation to oxidative stress are not yet fully understood.

Endothelial dysfunction was found in cardiovascular diseases, including hypertension [15–17]. Normal endothelium secretes vasoactive substances to regulate vascular tone, and endothelium-derived nitric oxide (NO) to maintain healthy vasculature. However, under oxidative stress, the bioavailability of NO is decreased; this is one of the hallmarks of endothelial dysfunction [18–22]. Increased reactive oxygen species (ROS) level during hypertension has been observed clinically and in vivo [20–22]. Certain anti-hypertensive medications, such as nebivolol and carvedilol, possess antioxidative activity to improve NO bioavailability [23–25].

This study aimed to investigate the effects of acupuncture on endothelial dysfunction in spontaneously hypertensive rats, and to determine the association of the observed changes in blood pressure, and biochemistry of endothelial function, NO bioavailability, and oxidative stress.

## Methods

### Animals and artery preparation

Eighteen week-old spontaneously hypertensive rats (SHRs) and Wistar-Kyoto rats (WKYs) were supplied by the Laboratory Animal Service Centre, The Chinese University of Hong Kong. Rats were kept in a holding room with temperature control of 22–24 °C and 12-h light/dark cycle. Standard diet and water were provided ad libitum. The animals were divided into four groups, including the WKY control group (n = 8), SHR control group (n = 8), SHR sham-acupuncture group (n = 8) and SHR real acupuncture group (n = 8). After 6 weeks of treatment, the animals were sacrificed by carbon dioxide inhalation. Aortas and carotid arteries were dissected out and the surrounding connective tissues were removed. This study was approved by the Animal Experimentation Ethic Committee (Additional file 1), The Chinese University of Hong Kong and conformed to the guidelines of the *Principles of Laboratory Animal Care* (NIH publications No. 80-23, revised 1996). The project proposals and proposal revised history were provided (Additional files 2, 3, 4). The members of clinical research ethics committee was provided (Additional file 5). The animal studies were performed following the ARRIVE guideline (Additional file 6).

### Acupuncture intervention

Electro-acupuncture was applied to SHRs in the SHR acupuncture group for 6 consecutive weeks. Acupuncture needles (13 mm in length and 0.14 mm in diameter) were inserted into the bilateral ST36 and LR3 acupoints. A regular manual manipulation of the needle was performed to elicit a gentle voluntary movement of the lower limbs of the rats, which was considered to be equivalent to the *deqi* (obtaining *qi*) sensation, typically felt by patients with the feelings of soreness, tingling, distension and heaviness. Needles were then connected to an electrical stimulator (Model E600 HAN Multi-Purpose Digital Electronic Acupunctoscope, Hong Kong), and the output of which was set to a continuous wave at 2 Hz, 400 μs band width with an intensity of 2 mA. The duration of each treatment was 20 min and acupuncture treatment was conducted once daily, 5 days every week for 6 consecutive weeks. In the SHR sham-acupuncture control group, intervention was adopted by pricking of a non-acupoint/non-meridian area using a blunted end needle without penetrating through the skin. The locations selected for the sham-acupuncture were over the posterolateral side of the calf muscle and lateral side of the feet approximately over the fifth metatarsal bone. The blunted end needle was removed immediately and no electric stimulation was applied; however, rats were restrained for 20 min as did in the acupuncture group. Both the SHR control and WKY control groups received no real or sham-acupuncture treatment during the 6-week period.

### Blood pressure monitoring

Blood pressure was non-invasively measured by determining tail blood volume with a volume pressure recording sensor and an occlusion tail-cuff (CODA System, Kent Scientific, Torrington, CT, USA). The trend of blood pressure was recorded at several time points, i.e., prior to treatment, at the 2nd, 4th week and the end of interventions [26].

### Measurement of isometric force

Aortic rings were suspended in organ bath filled with Krebs solution (pH7.4) containing 119 mM NaCl, 4.7 mM KCl, 2.5 mM CaCl_2_, 1 mM MgCl_2_, 25 mM NaHCO_3_, 1.2 mM KH_2_PO_4_, and 11 mM d-glucose, oxygenated with 95 % O_2_–5 % CO_2_, and kept at 37 °C. Aortic rings were fixed in one end to a metal hook and the other end connected to a force transducer under a basal tension of 25 mN as described previously [27]. Carotid arteries were suspended in wire myograph (Danish Myo Technology, Denmark) under a basal tension of 5mN. They were allowed to equilibrate for 30 min and then challenged by 60 mM KCl solution to ensure the viability of the aortas. Phenylephrine (0.3 μM) was used to induce a contraction followed by relaxation by acetylcholine (10 μM) for assessing the integrity of functional endothelium. After washout, phenylephrine (0.3 μM) was added again to produce a stable contraction, then acetylcholine (ACh) was applied cumulatively (3 nM–3 μM) to induce endothelium-dependent relaxations. In some rings treated with L-NAME (100 μM) for 30 min, endothelium-independent relaxations were examined in response to NO donor sodium nitroprusside (SNP).

### Western blot analysis

Aortic rings were frozen in liquid nitrogen and homogenized in ice-cold RIPA lysis buffer (1 mg/mL leupeptin, 5 mg/mL aprotonin, 100 mg/mL PMSF, 1 mM sodium orthovanadate, 1 mM EGTA, 1 mM EDTA, 1 mM NaF, and 2 mg/mL b-glycerolphosphate). The lysates were centrifuged (Centrifuge 5430R, EppendorfAG, Hamburg, Germany) at 20,000×*g* for 20 min at 4 °C. The supernatants were collected and the protein concentration was determined by the Lowry method (BioRad, CA, USA). Samples containing 20 μg of protein was boiled for 10 min with 5 % β-mercaptoethanol and then separated on a 10 % SDS–polyacrylamide gel by electrophoresis. The resolved protein was transferred to an immobilion-P polyvinylidenedifluoride membrane (Millipore Corp., Bedford, MA, USA) and blocked with 1 % BSA for 20 min. Primary antibodies against eNOS (BD, Lexington, KY, USA), nitrotyrosine (Upstate, Lake Placid, NY, USA) and GAPDH (Ambion, Austin, TX, USA) were used for incubation at 4 °C overnight, followed by horseradish peroxidase-conjugated secondary antibodies (DakoCytomation, Glostrup, Denmark). An enhanced chemiluminescence detection system (ECL reagents, Amersham Pharmacia Biotech, Buckinghamshire, UK) was applied to develop the membranes and a documentation program (Fluochem, Alpha innotect Corp., San Leandro, CA, USA) was used for densitometry measurement [28].

### Measurement of antioxidant capacity

After rat euthanasia, blood was collected and stored in tubes containing heparin. The samples were centrifuged at 1000×*g* for 10 min at 4 °C; plasma was then collected and stored on ice. The assay was performed using an antioxidant assay kit (Cayman Chemicals, Ann Arbor, MI, USA) according to the manufacturer’s instructions. The principle of the assay is based on the antioxidant activity in the sample to inhibit the oxidation of 2,2′-azino-di-[3-ethylbenzthiazoline sulfonate] by metmyoglobin. The antioxidant capacity was compared with that of Trolox, a water-soluble tocopherol analogue, as the standard, and the absorbance at 750 nm was measured colorimetrically (ELX800; BioTek Instruments, Winooski, VT, USA) [29].

### Measurement of serum angiotensin II level

After rat euthanasia, blood was collected and stored in tubes containing EDTA on ice. Blood samples were centrifuged at 3000×*g* for 20 min at 4 °C, and serum was extracted immediately. Angiotensin II level was assessed using an enzyme immunoassay kit (SPI-Bio, Massy, France) according to the manufacturer’s instructions [30].

### Measurement of NADPH oxidase activity

Aortic tissue was prepared and homogenized in lysis buffer as described above. The supernatant was used to measure the NADPH oxidase activity by a lucigenin chemiluminescence assay. We mixed 60 µg of sample with lucigenin (10 µM, final concentration) and NADPH (100 µM, final concentration) in a dark environment. The chemiluminescence signal was then measured every 1 min for 10 min using a luminometer (GloMax 20/20; Promega, Madison, WI, USA). An extra set of controls was designed by adding diphenyleneiodonium (DPI, 5 µM), a NADPH oxidase inhibitor, in the homogenate of the SHR control group [31].

### Measurement of aortic nitrite/nitrate level

Aortic rings were prepared and homogenized in lysis buffer as described above. The supernatant was used to measure the serum nitrite/nitrate level by colorimetric assay kit (Cayman Chemicals, Ann Arbor, MI, USA). We used 30 µg of sample for the measurement according to the manufacturer’s instructions [32].

### Measurement of reactive oxygen species by dihydroethidium imaging

Dihydroethidium (DHE; Molecular Probes, Eugene, OR, USA) was used to evaluate the amount of oxidant formation [33]. Frozen sections of aortic rings and carotid arteries were sectioned at 10 µm thickness by cryostat microtome (Leica CM1100; Leica Instruments, Germany) and incubated in Krebs solution containing 5 µM DHE for 10 min at 37 °C. Samples were examined with a confocal microscope (FV1000; Olympus, Tokyo, Japan) at an excitation/emission of 488/605 nm. Fluorescence intensity was quantified by Fluoview version 1.5 (FV10-ASW1.5; Olympus, Tokyo, Japan).

### Immunohistochemistry

Aortic rings were immunohistochemically stained. Aortas were fixed in 4 % paraformaldehyde overnight at 4 °C, and then processed and embedded in paraffin. Rings were cut into 5 μM cross-sections by microtome (Leica CM1100; Leica Instruments, Germany) and placed on glass slides. Slides were de-waxed, dehydrated, immersed in 5 % H_2_O_2_ in methanol, and boiled in 0.01 mol/L citrate buffer (pH 6.0) for 3 min in a microwave for antigen retrieval. Donkey serum (5 %; Jackson Immunoresearch, West Grove, PA, USA) was used to incubate the sections for 30 min for blocking. Primary antibody nitrotyrosine (1:500; Upstate, Lake Placid, NY, USA) diluted in donkey serum was used for overnight incubation at 4 °C. The slides were washed in phosphate-buffered solution (PBS) three times (5 min each time) and incubated with secondary biotin-SP conjugated goat anti-rabbit antibody (1:200; Jackson Immunoresearch, West Grove, PA, USA) for 1 h at room temperature. The slides were again washed three times in PBS, followed by incubation with streptavidin-HRP conjugate (ZymedLaboratories Inc., South San Francisco, CA, USA) at 1:200 dilution for 1 h at room temperature. Visualization was done by 3,3′-diaminibenzide tetrachloride (DAB) staining (Vector Laboratories Inc., Burlingame, CA, USA). DAB chromogen substrate was added and incubated for 2 min on the sections. Slides were rinsed in running water for 10 min and counterstained with hematoxylin. Dehydration and mounting were done and microscopic pictures were taken using Spot Advanced software (Diagnostic Instruments, Detroit, MI, USA). Images and intensity of signals were analyzed using ImageJ software (National Institutes of Health, Bethesda, MD, USA) [34].

### Histological study and connective tissue assessment

Glass slides with sections of aortic rings were prepared by fixation in paraffin, cutting in sections, de-waxing and dehydration as for immunohistochemistry stain. Sections were directly stained for collagen and elastin contents, and histological study. After de-waxing, the sections were stained using the Weigert–Van Gieson method [35] for collagen content assessment. Slides were immersed in 2.5 % lithium carmine, or named solution A (25 g carmine in 100 mL saturated solution of lithium carbonate), for 5 min and washed in water for 2 min. The slides were then placed in Weigert’s stain, or named solution B (basic fuchsin 2 g, resorcinol 4 g, distilled water 300 mL, 20 % ferric perchloride solution 40 mL, 95 % ethanol 280 mL, hydrochloric acid 2.8 mL), for another 5 min followed by washing in tap water for 2 min. Differentiation was carried out in 1 % acid alcohol, and sections were finally stained in Van Gieson’s stain (saturated aqueous picric acid 80 mL, 1 % acid fuchsin 20 mL) for 10 min. Slides were counterstained with hematoxylin and then washed, dehydrated and mounted for microscopic assessment of collagen content and histological studies. Elastin content was assessed by the orcein technique [36]; synthetic orcein (1 g) was dissolved in 100 mL of 70 % alcohol with the aid of heat, followed by cooling and filtering. Hydrochloric acid (1 mL) was added in the last step of stain preparation. Slides were placed in the stain for 1 h at room temperature. They were then rinsed in 70 % alcohol and differentiated in acid alcohol, followed by washing in tap water for 2 min. Counterstaining was carried out using hematoxylin before the slides were washed, dehydrated and mounted for further analysis. Collagen and elastin contents were quantified using ImageJ software.

Under the same section area and using identical magnification, the areas of collagen and elastin stain were measured and expressed as fold change relative to WKY control sections. For collagen and elastin fractions, the same length of segments were selected in sections and the areas of collagen or elastin stain were measured over the total selected area [37].

### Data analysis

Results were expressed as mean ± SD. The relaxation of aortic rings and carotid arteries was presented as percentage of the evoked tone. Data were analyzed using GraphPad Prism software (GraphPad Software Inc., San Diego, CA, USA). The cumulative concentration–response curve was analyzed with a non-linear curve fitting. The maximal relaxation response (E_max_) and negative logarithm of the dilator concentrations to cause 50 % relaxation (pD_2_) were calculated. The relaxation at different concentration of drugs was compared. Statistical analysis was performed by one-way ANOVA followed by the Newman–Keuls test for comparisons among all groups. *P* < 0.05 was considered statistically significant. Exact *P* values were shown, unless *P* < 0.001.

## Results

### Acupuncture attenuated blood pressure in spontaneously hypertensive rats

**Fig. 1:**
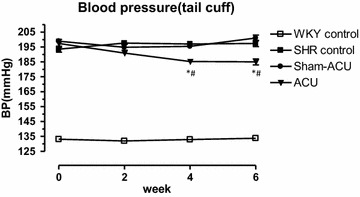
Effect of acupuncture treatment on blood pressure in experimental rats. Data were expressed as means ± SD. (*^,#^ *P* < 0.001 for ACU vs. SHR control and sham-acupuncture, respectively)

The blood pressure trends during 6 weeks of acupuncture treatment are shown in Fig. 1. At the beginning of the treatment, blood pressure was similar among the SHR control, SHR sham-acupuncture and SHR acupuncture groups (193.5 ± 5.8, 198.9 ± 4.5 and 197.4 ± 5.2 mmHg, respectively). Blood pressure in the WKY group was significantly lower than that in the SHR groups (133.1 ± 4.1 vs. 193.5 ± 5.8 mmHg, *P* < 0.001). At the end of treatment, blood pressure was significantly lower in the SHR acupuncture group (185.0 ± 5.6 mmHg) compared with the SHR sham-acupuncture group (201.0 ± 5.4 mmHg) and SHR control group (197.4 ± 5.9 mmHg; *P* < 0.001). There was a significant reduction in blood pressure in the SHR acupuncture group compared with the three control groups (Table 1).

**Table 1:** The blood pressures at the beginning, 4th and 6th week of treatment

	BP, mmHg (0 week)	BP, mmHg (4th week)	BP, mmHg (6th week)	Change in BP, mmHg
WKY control	133.1 ± 4.1	132.9 ± 4.0	133.8 ± 5.0	+0.6 ± 3.8
SHR control	193.5 ± 5.8	197.0 ± 4.6	197.4 ± 5.9	+3.9 ± 3.8
Sham-acupuncture	198.9 ± 4.5	195.4 ± 4.2	201.0 ± 5.5	+2.1 ± 4.0
Acupuncture	197.4 ± 7.5	185.3 ± 5.0*^,#^	185.0 ± 5.7*^,#^	−12.4 ± 6.3*^,#^

Data were expressed as means ± SD

*^, #^ *P* < 0.001 for ACU vs. SHR control and sham-acupuncture respectively

### Acupuncture improved endothelium-dependent relaxations in aortas and carotid arteries

**Fig. 2:**
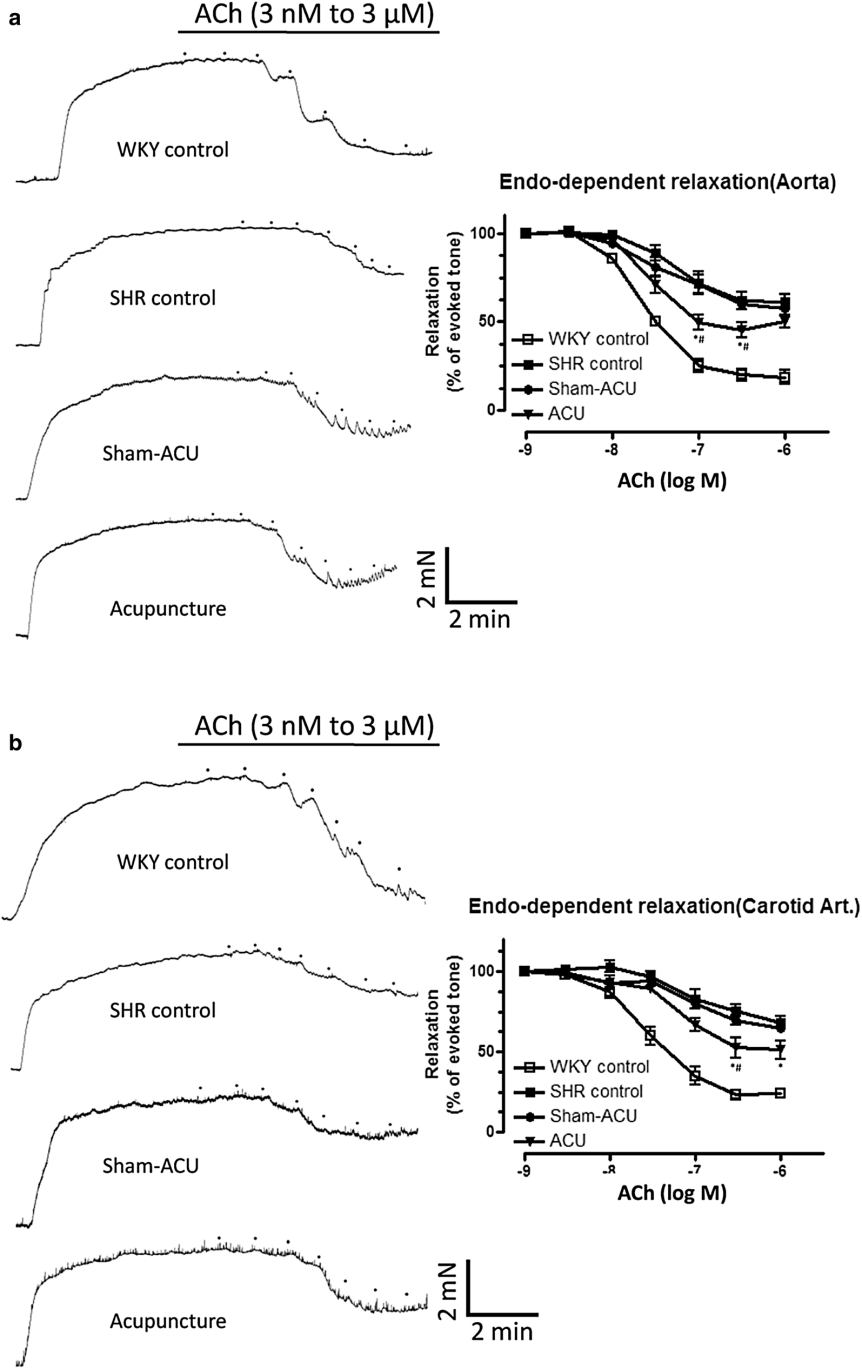
The cumulative concentration–response curves for Ach-induced relaxations of different groups in aorta (**a**) and carotid (**b**) arteries. Data were expressed as means ± SD. (For aorta at ACh[log M] concentration of −6.5,*P = 0.024 for ACU vs. SHR control and ^#^ *P* = 0.019 for ACU vs. sham-acupuncture. At ACh[log M] concentration of −7, *P = 0.018 for ACU vs. SHR control and ^#^ *P* = 0.0095 vs. sham-acupuncture. For carotid artery at ACh[log M] concentration of −6,*P = 0.045 for ACU vs. SHR control. At ACh[log M] concentration of −6.5, *P = 0.022 for ACU vs. SHR control and ^#^ *P* = 0.05 for ACU vs. sham-acupuncture)

Acetylcholine-induced endothelium-dependent relaxation (EDR) in the aortas was significantly lower in the SHR control group (E_max_% 38.5 ± 9.5) compared with the WKY control group (E_max_% 81.3 ± 7.9; *P* < 0.001). Sham-acupuncture did not affect EDR in the SHRs (E_max_% 42.5 ± 9.1). However, acupuncture treatment significantly improved EDR in the SHRs; E_max_% in the SHR sham-acupuncture control group (54.3 ± 10.1) was significantly lower than that in the SHR control group (E_max_% 42.5 ± 9.1; *P* = 0.0191) and the WKY control group (E_max_% 38.5 ± 9.5; *P* = 0.0238) (Fig. 2; Table 2).

**Table 2:** ACh-induced relaxations in the aorta and carotid arteries of the rats in the acupuncture and control groups

	pD_2_	E_max_ (%)
ACh relaxation of aortas		
WKY control	7.63 ± 0.22	81.3 ± 7.9
SHR control	7.27 ± 0.61	38.5 ± 9.5
Sham-acupuncture	7.36 ± 0.37	42.5 ± 9.1
Acupuncture	7.55 ± 0.83	54.3 ± 10.1*^,#^
ACh relaxation of carotid arteries		
WKY control	7.54 ± 0.29	76.7 ± 5.2
SHR control	7.04 ± 0.68	31.8 ± 7.3
Sham-acupuncture	7.00 ± 0.44	35.2 ± 4.3
Acupuncture	7.16 ± 0.54	48.9 ± 9.6*

Data were expressed as means ± SD. (*^,#^ refer to Fig. 2)

**Fig. 3:**
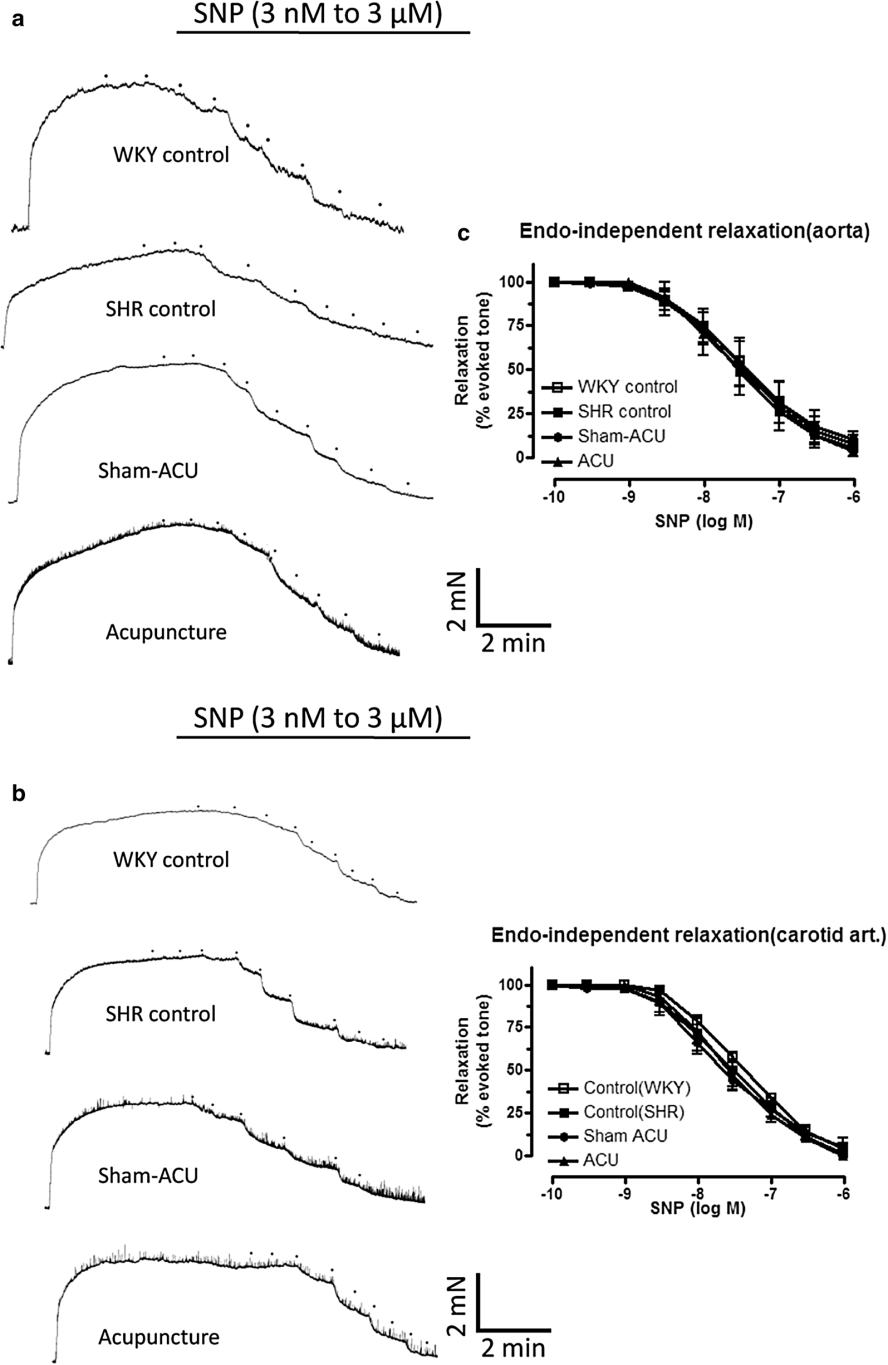
The cumulative concentration–response curves for the SNP-induced relaxations of different groups in aorta (**a**) and carotid (**b**) arteries. Acupuncture did not restore endothelium-independent relaxation (**c**). Data were expressed as means ± SD

There were similar findings in the carotid arteries. The EDR of the SHR control group was significantly lower than that of the WKY control group (*P* < 0.001). Acupuncture treatment partially restored EDR, while sham-acupuncture exerted no effect. Endothelium-independent relaxation was similar in all four groups (Fig. 3; Table 3).

**Table 3:** The SNP-induced relaxations in the aorta and carotid arteries of the rats in the acupuncture and control groups

Treatment	pD_2_	E_max_ (%)
SNP relaxation of aortas		
WKY control	7.47 ± 0.26	98.0 ± 19.8
SHR control	7.56 ± 0.18	97.7 ± 11.4
Sham-acupuncture	7.52 ± 0.26	93.3 ± 17.8
Acupuncture	7.51 ± 0.27	101.6 ± 23.1
SNP relaxation of carotid arteries		
WKY control	7.38 ± 0.07	100.3 ± 5.3
SHR control	7.48 ± 0.18	100.4 ± 16.1
Sham-acupuncture	7.63 ± 0.15	97.6 ± 8.8
Acupuncture	7.55 ± 0.15	101.8 ± 9.8

Data were expressed as means ± SD

### Acupuncture increased the eNOS expression and attenuated nitrotyrosine level

**Fig. 4:**
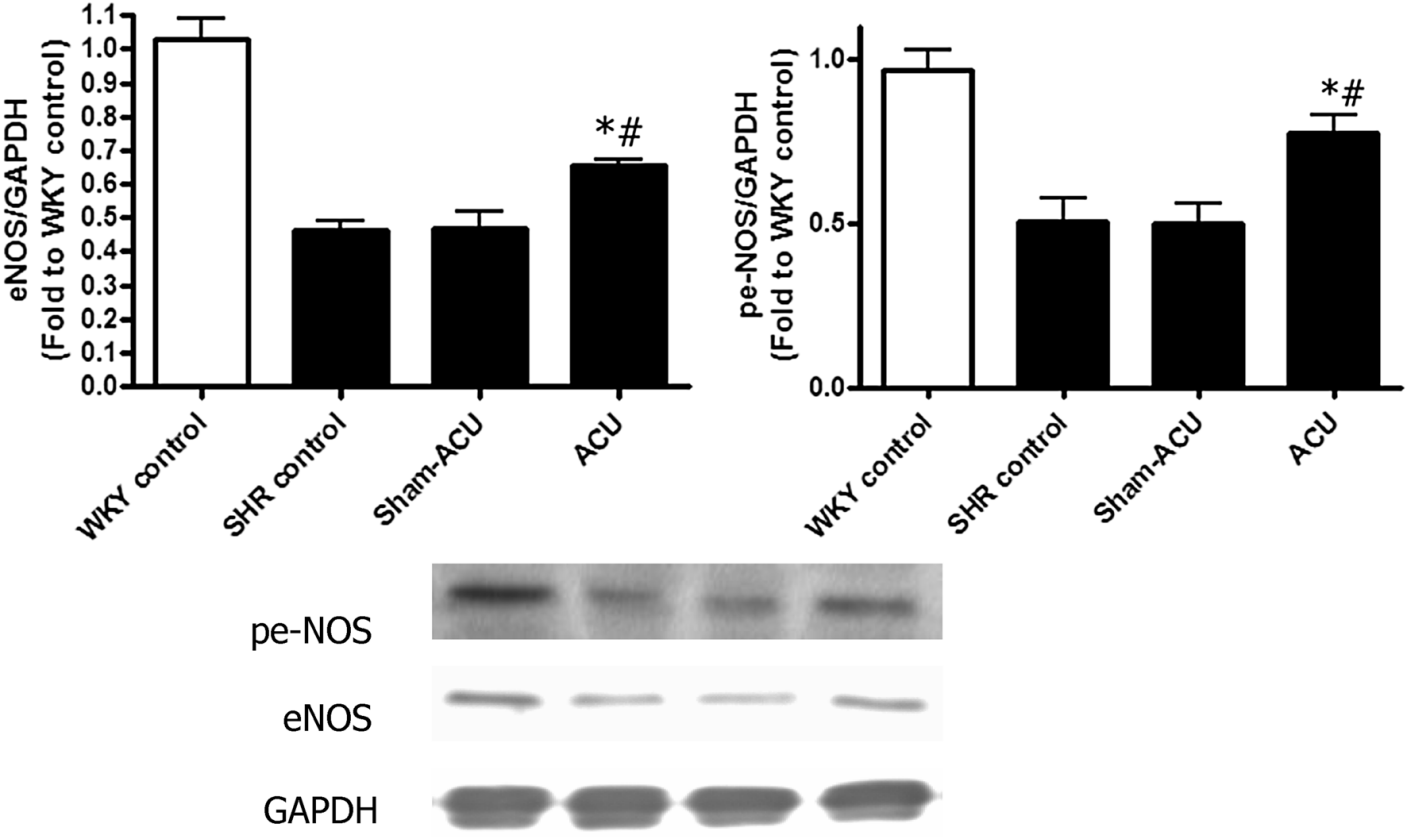
Effects of acupuncture on eNOS and peNOS expressions in the aorta tissues. Acupuncture significantly enhanced the eNOS and peNOS expressions when compared with the SHR control and sham-acupuncture group. Data were expressed as means ± SD. (For eNOS expression, *P < 0.001 for ACU vs. SHR control and ^#^ *P* = 0.0053 for ACU vs. sham-acupuncture. For peNOS expression, *P* = 0.012 for ACU vs. SHR control and ^#^ *P* = 0.0073 for ACU vs. sham-acupuncture)

**Fig. 5:**
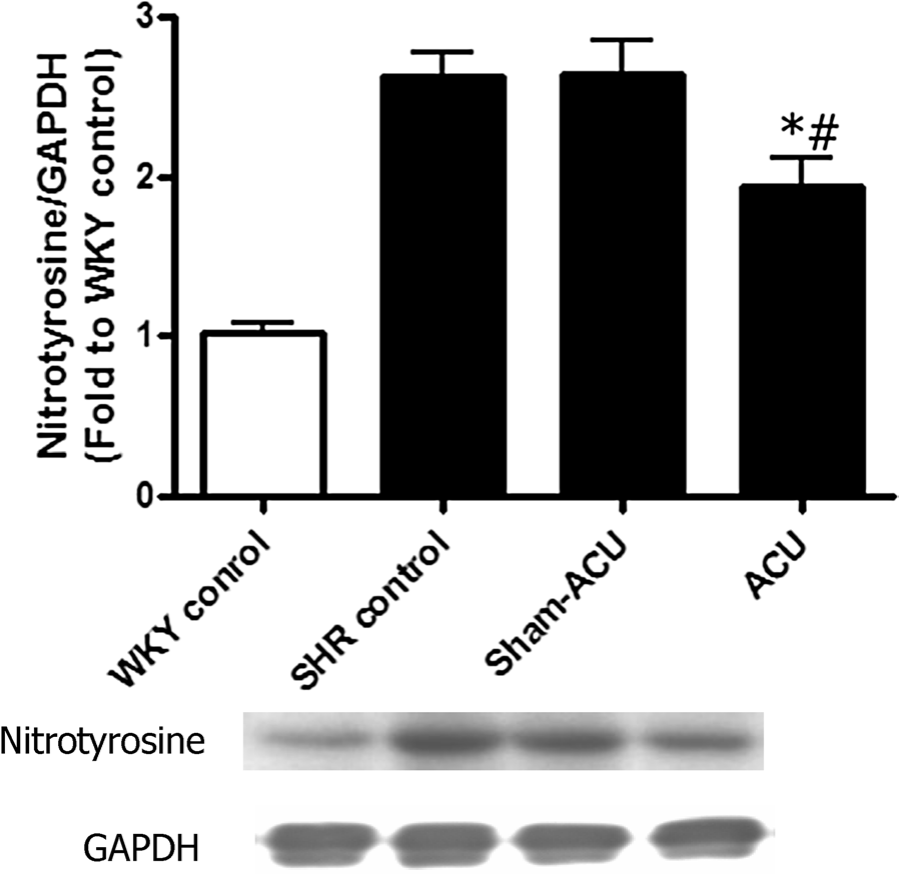
Effects of acupuncture on nitrotyrosine expression in the aorta tissues. Both the SHR control and sham-acupuncture group showed an increased nitrotyrosine expression when compared with the WKY control. Acupuncture treatment markedly inhibited nitrotyrosine expression. Data were expressed as means ± SD (*P = 0.0012 for the SHR vs. the WKY control and ^#^ *P* = 0.0073 for ACU vs. sham-acupuncture)

The eNOS expression in aortas of the SHR control group was significantly decreased, compared with the WKY control group (*P* < 0.001). Acupuncture significantly increased the eNOS expression (*P* < 0.001), but sham-acupuncture showed no effect. Nitrotyrosine was overexpressed in the SHR control group compared with the WKY control group; acupuncture, but not sham treatment, attenuated its expression (Figs. 4, 5).

### Acupuncture enhanced the antioxidant capacity in the aortas

**Fig. 6:**
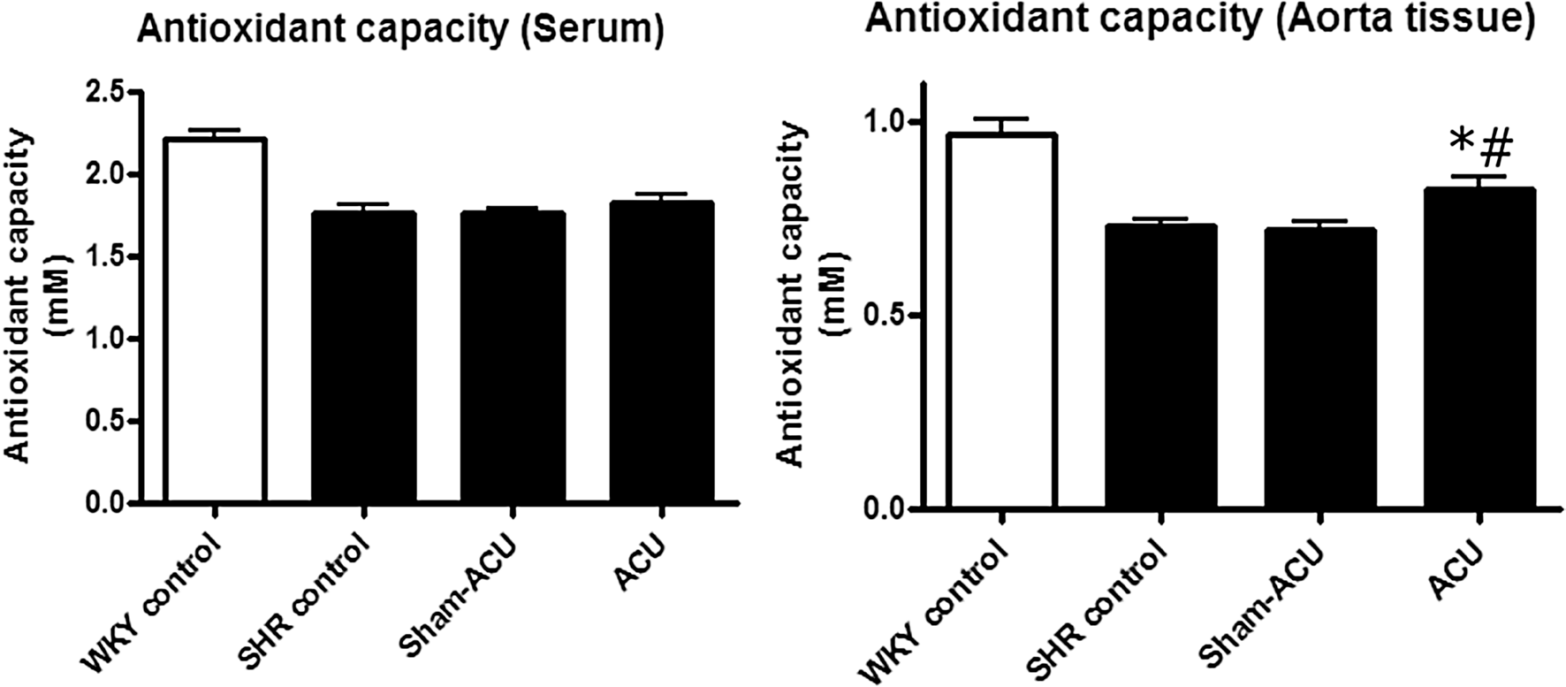
Antioxidant capacity of different groups. Antioxidant capacity was lower in both serum and aorta tissues of the SHR control when compared with SHR. The aorta tissue in the acupuncture group showed an improved antioxidant capacity. Data were expressed as means ± SD (In aorta tissue, *P = 0.0039 for ACU vs. SHR control and ^#^ *P* = 0.0032 for ACU vs. sham-acupuncture)

The serum of the WKY control group showed a significantly higher antioxidant capacity than that of the SHR control group (*P* < 0.001); neither sham-acupuncture nor acupuncture affected the antioxidant capacity. In aortic homogenate, the antioxidant capacity was significantly higher in the WKY control compared with the SHR control group (*P* < 0.001). Acupuncture promoted antioxidant capacity, while sham-acupuncture elicited no effect (Fig. 6).

### Acupuncture reduced serum angiotensin II level

**Fig. 7:**
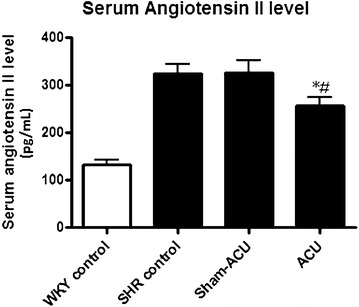
Effects of acupuncture treatment on serum angiotensin II level as measured by EIA kit. The level was significantly attenuated after acupuncture treatment when compared with the SHR control and sham-acupuncture group. Data were expressed as means ± SD (*P = 0.023 for ACU vs. SHR control and ^#^ *P* = 0.035 for ACU vs. sham-acupuncture)

As shown in Fig. 2, the serum angiotensin II levels were significantly higher in both the SHR control group (324.4 ± 57.9 pg/mL) and the SHR sham-acupuncture group (327.1 ± 71.1 pg/mL) compared with the WKY control group (132.0 ± 27.2 pg/mL; *P* < 0.001). For the SHRs receiving 6 weeks of acupuncture, the angiotensin II level was significantly reduced (256 ± 49.1 pg/mL) compared with the sham-acupuncture control (*P* = 0.036) and the SHR control group (*P* = 0.023; Fig. 7).

### Acupuncture attenuated NADPH oxidase activity in the aortas

**Fig. 8:**
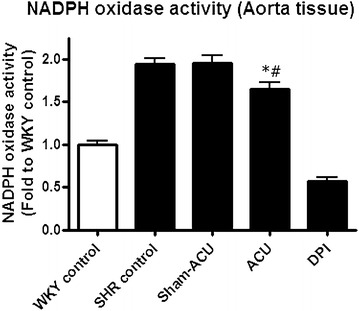
Effects of acupuncture on NADPH oxidase activity. NADPH oxidase activity in the WKY control was lower than that in the SHR control. Sham-acupuncture did not affect the enzyme activity but real acupuncture significantly reduced it. DPI, a NADPH oxidase inhibitor that can markedly inhibit the enzyme’s activity. Data were expressed as means ± SD (*P = 0.022 for ACU vs. SHR control and ^#^ *P* = 0.028 for ACU vs. sham-acupuncture)

The enzymatic activity was significantly lower in the WKY control group compared with the SHR control group (*P* < 0.001). Acupuncture on the SHRs significantly reduced NADPH oxidase activity in the aorta (*P* = 0.022), while sham-acupuncture exerted no effect. An extra control group created by incubating the aortas with DPI showed a markedly reduced NADPH oxidase level in tissue homogenate (*P* < 0.001) (Fig. 8).

### Acupuncture increased aortic nitrite/nitrate level

**Fig. 9:**
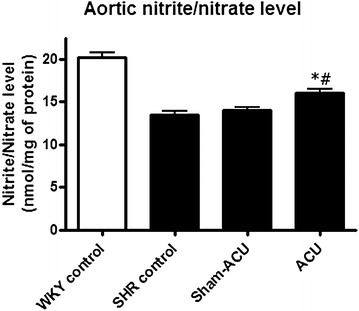
Effects of acupuncture treatment on aortic nitrite/nitrate level. The level was significantly accentuated after acupuncture treatment when compared with the SHR control and sham-acupuncture group. Data were expressed as means ± SD (*P = 0.0018 for ACU vs. SHR control and ^#^ *P* = 0.0061 for ACU vs. sham-acupuncture)

Nitrite/nitrate level was significantly higher in the WKY control group compared with the SHR control group (*P* < 0.001). Acupuncture on the SHRs attenuated the nitrite/nitrate level, while sham-acupuncture produced no effect (Fig. 9).

### Acupuncture reduced reactive oxygen species in the aortas and carotid arteries

**Fig. 10:**
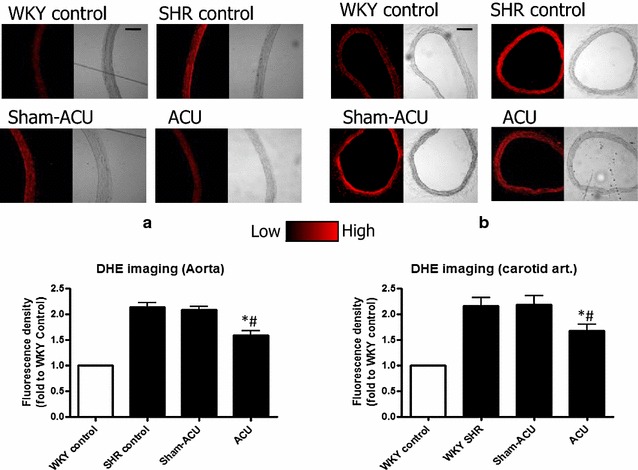
ROS accumulation in aorta (**a**) and carotid arteries (**b**) as detected by DHE staining. Acupuncture treatment significantly lowered the ROS level in aorta. (*P = 0.0017 for ACU vs. SHR control and ^#^ *P* = 0.0016 for ACU vs. sham-acupuncture.) and carotid arteries (*P = 0.039 for ACU vs. SHR control and ^#^ *P* = 0.0449 for ACU vs. sham-acupuncture.). Data were expressed as means ± SD

DHE fluorescence in the aortas was significantly elevated in the SHR control and the SHR sham-acupuncture group compared with the WKY control group (*P* < 0.001) (Fig. 10). Acupuncture treatment was able to mitigate aortic ROS production. Similar results were observed in carotid arteries.

### Acupuncture reduced nitrotyrosine expression in the endothelial layer

**Fig. 11:**
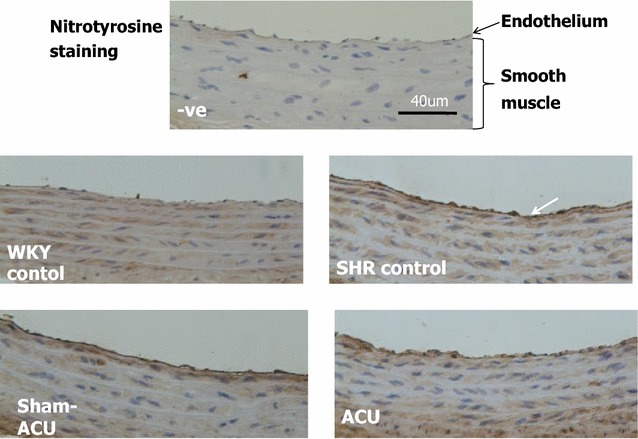
Immunohistochemistry localization of nitrotyrosine expression in aorta tissues. The negative (−ve) control used no nitrotyrosine antibodies and yielded no staining of tissues. In the SHR control and sham-acupuncture group, a clear staining of the intima was observed. In the acupuncture group, the intensity of the staining was attenuated, while the WKY control group only showed a weak signal

The nitrotyrosine immunostaining was weak in the aortas of the WKY control group, while a prominent stain was observed in the intima of the SHR control and the SHR sham-acupuncture group. The intensity of staining was attenuated in the SHR acupuncture group (Fig. 11).

### Acupuncture exerted no effect on vascular structure and connective tissues

**Fig. 12:**
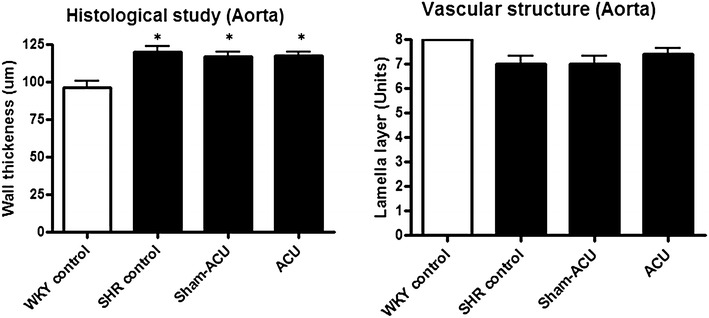
Effects of acupuncture on vascular structure. The WKY rats had a thinner aorta wall than the SHRs and acupuncture exerted no effect on wall thickness. The number of lamella layer showed no difference among all four groups (*P < 0.001 for the WKY control vs. all other groups)

The WKY control group had a thinner intima-media compared with that of the SHR groups; both sham-acupuncture and acupuncture had no effect on this. There was no statistical difference in the thickness of the lamella layer between the SHR acupuncture group, the SHR sham-acupuncture group, and the SHR control group (*P* = 0.56) (Fig. 12).

**Fig. 13:**
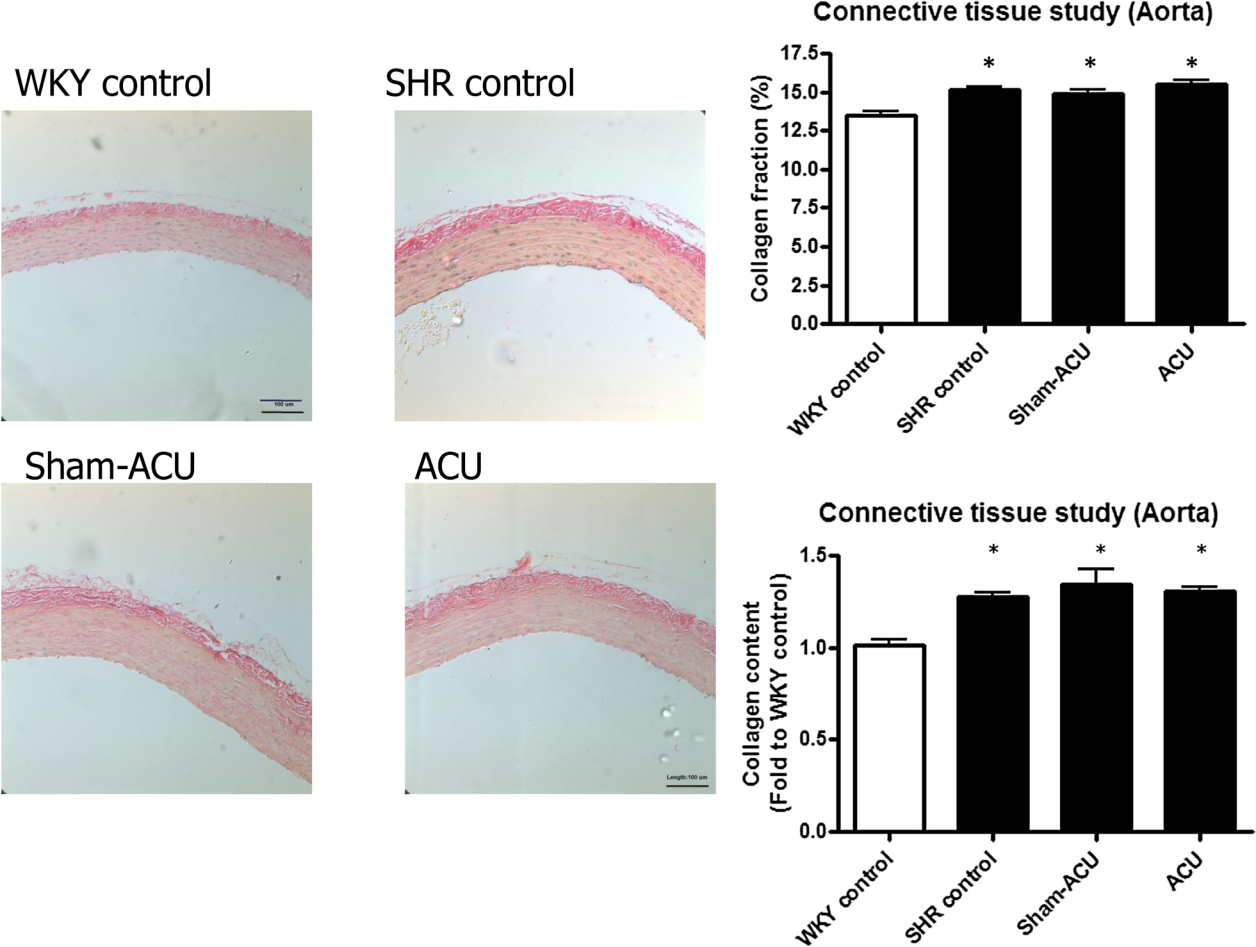
Effects of acupuncture on collagen content. Collagen was stained in red by the Van Gieson’s stain. The total collagen and fraction of collagen were lower in the WKY control. Acupuncture did not affect the collagen content. Data were expressed as means ± SD (*P < 0.001 for the WKY control vs. all other groups)

There was significantly more total collagen in the aortic ring sections of the SHRs than in the WKYs (*P* < 0.001). Both the SHR sham-acupuncture and SHR acupuncture groups had a similar amount of total collagen to the SHR control group (*P* = 0.59). The SHRs also had a significantly higher fraction of collagen compared with the WKYs (*P* < 0.001). Neither sham-acupuncture nor acupuncture affected the collagen fraction compared with the SHR control group (*P* = 0.22) (Fig. 13).

**Fig. 14:**
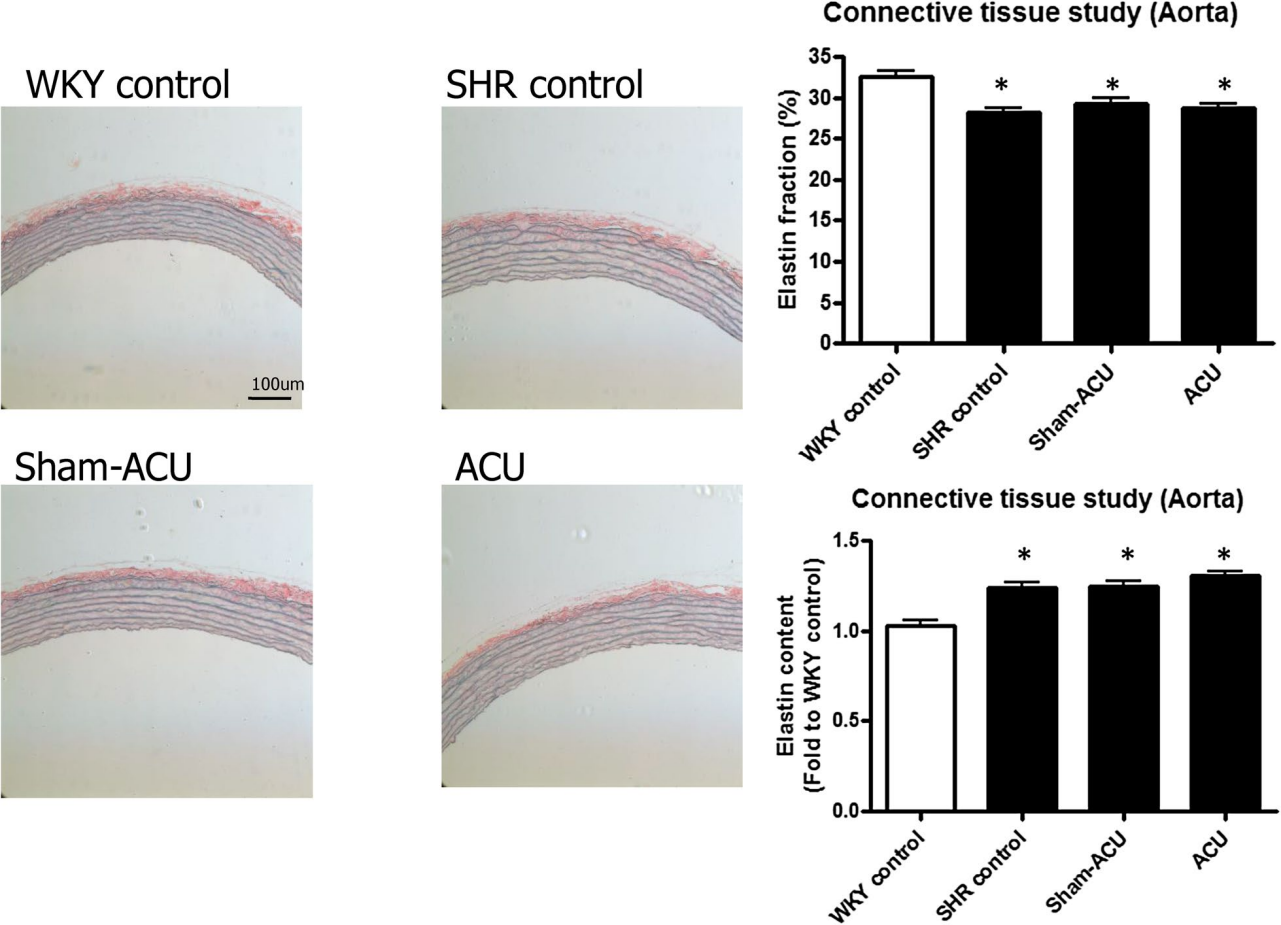
Effects of acupuncture on elastin content. Elastin was stained blue by Orcein stain. The WKY control had a higher proportion of elastin but the total content of it in their aorta tissues was lower. Acupuncture and sham-acupuncture showed no effect on both elastin fraction and elastin content. Data were expressed as means ± SD (*P < 0.001 for the WKY control vs. all other groups)

The elastin amount in the WKYs was significantly lower compared with that in the SHRs (*P* < 0.001). No difference was observed in the SHR sham-acupuncture and the SHR acupuncture group compared with the SHR control group (*P* = 0.23). The elastin fraction was significantly higher in the WKY control group than in the SHR control group (*P* < 0.001). The elastin fraction in the SHR sham-acupuncture and SHR acupuncture groups was similar to that in the SHR control group (*P* = 0.44) (Fig. 14).

### Acupuncture exerted no effects on vascular inflammation

**Fig. 15:**
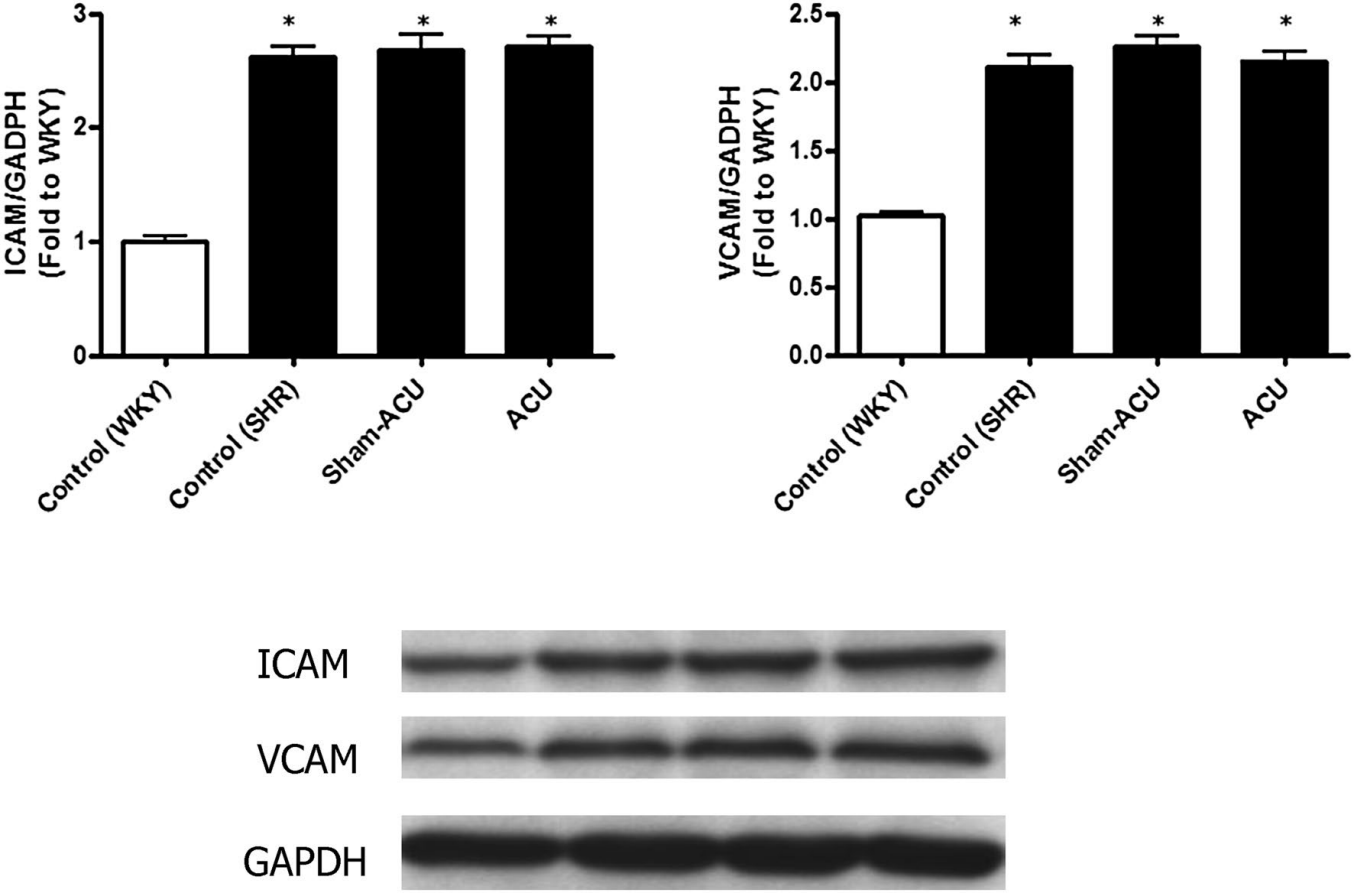
Effects of acupuncture on the ICAM and VCAM expressions in the aorta tissues. The expressions of these inflammatory markers were lower in the WKY control. Sham-acupuncture and real acupuncture did not affect the level of ICAM and VCAM expression. Data were expressed as means ± SD (*P < 0.001 for the WKY control vs. all other groups)

The effects of acupuncture on vascular inflammation were studied by measuring the intercellular adhesion molecule (ICAM) and vascular cell adhesion molecule (VCAM) expression by Western blot analysis. The WKY control group showed a significantly lower expression of both ICAM and VCAM compared with the SHR groups (*P* < 0.001); both sham-acupuncture and real acupuncture had no effect (Fig. 15).

### Adverse effects and complications

No morbidity or mortality was observed in the rats during the course of acupuncture and sham-acupuncture treatment. In addition, there was no abnormality of lower limb movement in any animal while receiving acupuncture treatment; only occasional minimal bleeding from the acupuncture site was seen, which was stopped by gentle manual pressure.

## Discussion

Effective treatment to lower high blood pressure can reduce the risk of stroke and myocardial infarction by 40 and 15 % respectively [38]. Anti-hypertensive medications remain the mainstay of treatment, in addition to lifestyle modifications such as weight reduction, physical exercise, cessation of smoking, and adoption of healthy diet. A meta-analysis [39] on the effect of acupuncture on essential hypertension in humans reported that acupuncture without antihypertensive medications did not significantly improve blood pressure in hypertensive patients; however, the number of studies included was small, and each study had a relatively small sample size. Another review [40] showed that acupuncture could lower systolic and diastolic blood pressure in humans, but the results were limited by the unsatisfactory methodological quality of the identified trials.

Although the search is still ongoing for solid evidence regarding the essence of the meridian system, increasing clinical data has been accumulated to support the efficacy of acupuncture [41–43]. In 2003, a document published by the WHO stated that acupuncture is suitable for treating primary hypotension and early essential hypertension [1]. The influence of acupuncture on hypertension might be related to its regulatory effect on the level of serum NO [1]; for mild and moderate essential hypertension, the hypotensive effect of acupuncture was more potent than placebo, and was comparable to certain conventional hypotensive agents [1]. At present, the evidence-based support for acupuncture as a treatment for hypertension in humans is still inconclusive. There has been no review on the effect of acupuncture on hypertension in animals, but many studies have reported significant attenuation of blood pressure [8, 44–48].

NO regulates blood vessel remodeling and angiogenesis [49, 50], and is also a major endothelium-derived relaxing factor for maintenance of normal vascular tone. In endothelial dysfunction, the bioavailability of NO is compromised, resulting in increased vascular tone and resistance [51]. Oxidative stress is closely associated with the bioavailability of NO, as free radicals may directly scavenge NO and uncouple its production enzyme, NOS [52]. Angiotensin II is a potent vasoconstrictor, and also acts as an important hormone to promote fluid retention; it may also increase oxidative stress by upregulating the activity of NADPH oxidase to produce more ROS [53, 54].

Endothelial dysfunction of the vessels is well known in 18-week-old SHRs [55] According to acupuncture theory, there are many acupoints that can be used to treat hypertension; among them, ST36 and LR3 are two of the most commonly used acupoints that have demonstrated effectiveness in lowering blood pressure in animal studies [8, 44–46]. In electroacupuncture, different frequency and wave types have previously been shown to have positive results in managing hypertension [8, 47, 48].

The regimen in our study of using a continuous wave at 2 Hz is a commonly used setting in acupuncture treatment of both humans and animals. In humans, blinding of treatment allocation can be achieved by using different devices such as Streitberger’s needle or Park’s sham device [56, 57]. However, in animal studies, the blinding of the animals from knowing whether they are receiving real acupuncture is less critical. The pricking of non-meridian non-acupoint locations by a blunt-ended needle can avoid the non-specific effect of needle penetration as well as the possible acupressure effect. In our study, there was no significant difference between the SHR control group that received no treatment and the SHR sham-acupuncture group.

In functional studies of aortas and carotid arteries, acupuncture improved the EDR but not the endothelial-independent relaxation in SHRs. eNOS is the NO-producing enzyme, and phosphorylated eNOS is the activated form of eNOS. The levels of both eNOS and phosphorylated eNOS were decreased in the SHRs compared with the WKYs. These assays could indirectly reflect the amount of NO in the vessel, and both expressions were promoted by real acupuncture treatment but not by sham-acupuncture. NO has a short half-life of only 4 s, and it undergoes a series of reactions in vivo to yield nitrite (NO_2−_) and nitrate (NO_3−_) [58]. As the proportion of these compounds varies in different physiological environments, we measured the total nitrite/nitrate level to assess the NO bioavailability. Aortic nitrite/nitrate levels of the SHRs were lower than that in the WKYs, and acupuncture treatment was able to further promote this reduction. Combined with the findings from the functional studies and protein blotting, it seemed that the beneficial effect of acupuncture on vascular reactivity was elicited via the enhancement of NO bioavailability.

The antioxidant capacity in aortic tissue homogenate was lower in the SHRs than in the WKYs, and acupuncture was able to promote this antioxidant capacity. The DHE signal from the aorta and carotid artery was stronger in the SHRs than the WKYs, suggesting that a higher level of superoxide anion was present in the vessels of the SHRs. The samples from the SHR acupuncture group revealed that the superoxide level was attenuated by acupuncture treatment. As it is difficult to measure peroxynitrite (i.e., oxidize lipoproteins or nitrate tyrosine residue of proteins), nitrotyrosine was used to indirectly indicate the peroxynitrite level and oxidative stress level. Western blotting revealed a significantly higher expression of the nitrotyrosine level in the SHRs than in the WKYs. Real acupuncture, not sham-acupuncture, attenuated nitrotyrosine expression.

Immunohistochemistry assays were performed to localize the nitrotyrosine. An increased intensity was clearly shown along the endothelial lining in the SHRs compared with the WKYs; a lighter staining was observed in the aortic ring from the SHR acupuncture group. Our results indicate that acupuncture was able to increase NO bioavailability in the rat vessels, and relieve oxidative stress.

The NADPH oxidase activity was higher in the SHRs than the WKYs, and acupuncture could attenuate this. As angiotensin II is one of the factors that stimulates NADPH oxidase, we also measured the serum angiotensin II and found that acupuncture treatment was able to reduce its level. It may be concluded that acupuncture treatment reduces blood pressure through altering a number of pathophysiological actions including fluid retention, vascular tone, NADPH oxidase activity, and oxidative stress.

Apart from the endothelium, acupuncture did not exert a significant effect on vascular structure in terms of wall thickness, number of lamellar layers, and the collagen and elastin content. Previous studies reported a reduced vessel wall thickness after acupuncture [59, 60], but we failed to reproduce similar results. This discrepancy could be owing to the difference in the experimental settings, including animal model, acupuncture technique, and treatment duration. Lastly, we investigated the anti-inflammatory effect of acupuncture, and observed a higher level of both ICAM-1 and VCAM-1 expression in the aortic tissues of the SHRs compared with that of the WKYs; however, both sham-acupuncture and acupuncture exerted no effect on ICAM-1 and VCAM-1 expression. This is the first animal study to demonstrate the close relationship between the reduction in oxidative stress and improvement in bioavailability of nitric oxide by acupuncture. However, the effects of acupuncture on other pathways related to the reactive oxygen species production clearly warrants further investigation. Moreover, the remodulation of other endothelial derived vasoactive substances and their mechanisms remained largely obscure. Our experimental findings provides a backbone for the investigation of the therapeutic action of acupuncture on hypertensive animals and the findings can help put the clinical use of acupuncture for hypertension on a solid scientific footing.

## Conclusion

Acupuncture treatment attenuated blood pressure in the SHRs. The effects of acupuncture in treating hypertension in the SHRs was associated with improvements in oxidative stress, NO bioavailability, and endothelial function.

## Additional files

10.1186/s13020-016-0110-0 Animal experimentation ethics approval.
10.1186/s13020-016-0110-0 Animal experimentation ethics committee.
10.1186/s13020-016-0110-0 Long-term experimentation.
10.1186/s13020-016-0110-0 Standard operating procedure.
10.1186/s13020-016-0110-0 The Joint CUHK-NTEC Clinical Research Ethics Committee.
10.1186/s13020-016-0110-0 The ARRIVE guidelines checklist.

## Abbreviations

Ach: acetylcholine
DHE: dihydroethidium
DPI: diphenyliodonium
EDCF: endothelial-derived constricting factor
EDHF: endothelial-derived hyperpolarizing factor
EDR: endothelial-dependent relaxation
EDRF: endothelial-derived relaxing factor
Emax: maximal relaxation response
eNOS: endothelial nitric oxide synthase
ICAM: intracellular adhesion molecule
L-NAME: NG-nitro-l-arginine methyl ester
NADPH: nicotinamide adenine dinucleotide phosphate
NO: nitricoxide
NOS: nitric oxide synthase
PBS: phosphate buffered saline
pD2: dilator concentrations to cause 50 % relaxation
peNOS: phosphorylated endothelial nitric oxide synthase
Phe: phenylephrine
ROS: reactive oxygen species
SDS-PAGE: sodium dodecyl sulfate polyacrylamide gel by electrophoresis
SHR: spontaneously hypertensive rat
SNP: sodium nitroprusside
SOD: superoxide dismutase
VCAM: vascular adhesion molecule
WKY: Wistar Kyoto rat
