# Supplementary material for: Attenuation of blood pressure in spontaneously hypertensive rats by acupuncture was associated with reduction oxidative stress and improvement from endothelial dysfunction
Source: Chin Med. 2016 Aug 30;11(1):38. doi: 10.1186/s13020-016-0110-0 (PMC5006281; doi:10.1186/s13020-016-0110-0)
Supplement: Supplementary file 2 — 10.1186/s13020-016-0110-0 Animal experimentation ethics committee. [file 13020_2016_110_MOESM2_ESM.doc]

# Animal Experimentation Ethics Committee (AEEC)

**
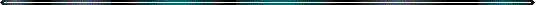
**

###### Application Form For Research

Proposal for use of experimental animals for research purposes at the Chinese University of Hong Kong

|  | Official Use |
| --- | --- |

Project Title:

| Acupuncture for Hypertension: A Mechanistic Study in Experimental Animals |
| --- |

1. Summary of proposal

A description of the rationale, objectives and methodology of the proposal (copy of full grant proposal is not acceptable).

| Hypertension is a chronic medical condition characterized by the elevated arterial blood pressure. It is defined as the systolic pressure exceeds 140 mmHg and/or diastolic pressure exceeds 90 mmHg. Hypertension is one of the major risk factors for heart attacks, cardiac failure, cerebrovascular accident and particularly chronic renal failure. The usual treatment of it included dietary control, execise advise, relaxation exercise and anti-hypertensive medications. But the use of complementary and alternative medicine has become increasingly popular in recent decades. Acupuncture is one of the traditional Chinese medicine techniques which has been widely practiced in many parts of the world to treat various kinds of diseases. Systematic reviews of clincal trials has shown that acupuncture can reduce blood pressure. World Health Organisation (WHO) has also recognised it as an effective treatment modality againist hypertension.  However, it remains poorly understood concerning the mechanisms of acupunture in lowering blood pressure. It is important to study its mode of action so as to fully utilize this cost-effective, easy-to-use and safe treatment modality. Previous animal studies have shown that acupuncture affect blood pressure mainly through its actions on the nervous and endocrine systems. The activty of certain parts of brain, the vagus and sympathetic nervous systems were found to be altered by acupuncture. In addition, endocrine system particularly the renin-angiotensin aldosterone cascade has been found to be inhibited by acupuncture. In the blood vessels, acupuncture was found to have a regulatory effect on endothelial-dervied relaxation and contracting factors such as nitric oxide, endothelin-1, and prostacyclin. Alterations in vascular structure such as the wall thickness and lumen diameter, VSMCs proliferation and apoptosis were also found after acupuncture treatment. Moreover, endothelial dysfuncton and vascular remodelling are the cardinal pathophysiology of blood vessels in hypertension. It is therefore important to study the effect of acupuncture on these pathological processes. |
| --- |

2. Academic and other staff involved in the experimental procedures (see Useful Information, item 6)

a.

|  | Name | Licence No. |
| --- | --- | --- |
|  | Professor Lin Zhi-xiu | pending |
|  | Mr. Leung Sin Bond | (10-335) in DH/HA&P/8/2/1 pt. 13 |
|  |  |  |
|  |  |  |
|  |  |  |
|  |  |  |
|  |  |  |
|  |  |  |
|  |  |  |
|  |  |  |

*****Please attach a copy of current animal licences for above workers*****

b. Brief description of experience of staff named in 2a with regard to animal experimentation.

| Professor Lin Zhi-xiu taught pharmacology to undergraduate students while working at Macau University of Science and Technology in 2003. Since joining CUHK in 2003, Prof. Lin has been involved in conducting animal experiments and supervising MPhil/PhD students' projects using animal models. The animal experiments conducted include establishment of an accute pancreatitis animal model, a xenograft pancreatic cancer model, a SCID-psoriasis model and a mouse tail test model for evaluation of anti-psoriatic drugs. Prof. Lin has extensive experience in animal handling and experimentation.  Mr. Leung Sin Bond holds a BSc degree in Biomedical Science from CUHK. He has extensive practical skills in animal handling and experimentation. |
| --- |

3. Advancement of scientific / medical knowledge

A clear explanation of how this project advances scientific / medical knowledge, and a clear indication that alternatives to experiments on live animals have been considered and why such alternatives were rejected.

| Through this project, we will be in a position to evaluate the effectiveness of acupuncture for primary hypertension. Moreover, the findings of this project should shed mechanistic insight into why acupuncture commoly used in Chinese medicine practice is efficacious for hypertension. The findings of the project would likely place the use of acupuncture for the treatment of hypertension on a scientific footing. Currently there is no suitable hypertension disease in vitro model e.g. cell lines and organ culture that mimic the true clinical features of primary hypertension, and the animal model proposed in this project, therefore, become indispensable for evaluation of acupuncture intervention. |
| --- |

4. Types of experiments to be performed

Indicate the types of experiments that you propose to perform on the appropriate form. Use a separate form for each species used. *(Please click the appropriate box)*

| Short-term | Form 1 | Euthanasia only |  |
| --- | --- | --- | --- |
|  | Form 2 | Experiments performed wholly under anaesthesia followed by termination of the animals |  |
|  | Form 3 | Experiments on conscious animals followed by termination of the animals |  |
| Long-term | Form 4 | Experiments on conscious animals |  |
|  | Form 5 | Experiments on conscious animals with period(s) of anaesthesia |  |

* Short-term experimentation means all procedures to be completed within one working day. In short-term experiments, there is no requirement for overnight supervision / care of the animal(s).

5. Hazards to personnel

Applicant must complete the form “Health, Safety & Environmental Assessment for Research Grant Applications” and submit details of the project for a full safety evaluation by the University Safety and Environment Office (USEO). However, at this stage, AEEC requires a brief indication of potential serious hazards posed by any of the treatments specified in this application (See Useful Information, Item 9).

| No potential hazards are anticipated for the experiments. |
| --- |

6. Declaration

I have read and agree to abide by the Animal (Control of Experiments) Ordinance Cap 340 and the AEEC Guidelines.

| **Investigator Names** | **Signature** | **Date** |
| --- | --- | --- |
| Professor Lin Zhi-xiu |  | August 9, 2010 |
| Mr Leung Sin Bond |  | August 9, 2010 |
|  |  |  |
|  |  |  |
|  |  |  |
|  |  |  |
|  |  |  |
|  |  |  |
|  |  |  |
|  |  |  |

7. Acknowledgement by Department Chairman / School Director

| **Name** | **Signature** | **Department / School** | **Date** |
| --- | --- | --- | --- |
| Professor Che Chun-Tao |  | School of Chinese Medicine | August 9, 2010 |

# 
