# Supplementary material for: Attenuation of blood pressure in spontaneously hypertensive rats by acupuncture was associated with reduction oxidative stress and improvement from endothelial dysfunction
Source: Chin Med. 2016 Aug 30;11(1):38. doi: 10.1186/s13020-016-0110-0 (PMC5006281; doi:10.1186/s13020-016-0110-0)
Supplement: Supplementary file 3 — 10.1186/s13020-016-0110-0 Long-term experimentation. [file 13020_2016_110_MOESM3_ESM.doc]

|  | Official Use |
| --- | --- |

##### LONG-TERM EXPERIMENTATION

**FORM 4**

**Experiments on conscious animals.**

Full details of dosing and tissue (e.g. blood) collection regimens must be given.

| A. | Brief description of experimentation | | | | Animals will be randomly divided into three groups with 10 of each: Group 1: Intervention group which will receive real acupuncture treatment;  Group 2: Sham acupuncture group which will receive sham acupuncture (acupuncture without penetration);  Group 3: Non-treatment control group.  For acupuncture treatment, animals will be stabilized with a specially designed restrainer. Acupuncture needles (Hwato brand, 0.26 mm in diameter, 13 mm in length stainless steel needle) will be inserted into the selected acupoints (bilateral ST36, LR3, and LI11) according to “Animal Acupoint guide”. The handle of needles will be connected to the Electric stimulator (model KWD-880II, ShenZhen electronic, China) using continuous wave, 2 Hz and 1 mA as output setting. Electric stimulation will be given for 30 min for each treatment session. Every 5 days treatment will be followed by 2 days rest. The total treatment duration is 3 weeks. |
| --- | --- | --- | --- | --- | --- |
| B. | Animals | | | |  |
|  | - | Species | | | Wistar kyoto rats, spontaneous hypertensive rats, Sprague-Dawley (SD) rats, IDDM mice and wild-type mice (C57 strain). |
|  | - | Sex | | | Male and female in equal ratio |
|  | - | Age | | | about 200 g for rats and 25 g for mice |
|  | - | Special characteristics | | | Nil |
|  | - | Total number and justification (e.g. providing group size, number of groups, number of experiments etc.) | | | Total 300 animals :  60 Wistar kyoto rats, 60 spontaneous hypertensive rats, 60 Sprague-Dawley (SD) rats, 60 IDDM mice and 60 wild-type C57 mice.  The experiment will be performed on different types of animals. Animals will be randomly divided into 3 groups with 10 of each: Group 1: Intervention group which will receive real acupuncture treatment;  Group 2: Sham acupuncture group which will receive sham acupuncture (acupuncture without penetration);  Group 3: Non-treatment control group.  The experiments will be performed twice. (3 x 10 x 2) x 5 species = 300 animals. |
| C. | Location | | | |  |
|  | - | Prior to experimentation | | Bldg | Laboratory Animal Services Centre, CUHK |
|  |  |  | | Room |  |
|  | - | During experimentation | | Bldg | Centralized Science Laboratory Building, CUHK |
|  |  |  | | Room |  |
| D. | Procedures  *List as 1, 2, 3 etc.*  *Do not include procedures performed after death of animal. Indicate total duration of procedures.* | | | | 1. Animals will be received real or sham acupuncture treatment for a total of 15 sessions.    2. For acupuncture treatment, animals will be stabilized with a specially designed restrainer. Acupuncture needles (Hwato brand, 0.26 mm in diameter, 13 mm in length stainless steel needle) will be inserted into the selected acupoints (bilateral ST36, LR3, and LI11) according to “Animal Acupoint guide”. The handle of needles will be connected to the Electric stimulator (model KWD-880II, ShenZhen electronic, China) using continuous wave, 2 Hz and 1 mA as output setting. Electric stimulation will be given for 30 min for each treatment session. Every 5 days treatment will be followed by 2 days rest. The total treatment duration is 3 weeks.  3. The arterial blood pressure will be measured by non-invasive tail-pulse cuff method several times a day to monitor the changes of blood pressure.  4. The animals will be sacrificed once the treatment cycle has been completed. |
| E. | Animal Welfare | | | |  |
|  | - | How often will the animals be monitored for their well-being? | | | 3 times per week |
|  | - | What parameters will be used to monitor and ensure the well-being of the animals?  (e.g. certain % loss of body weight, obvious distress, extent of tumour growth) | | | The animals will be monitored on their body weight loss (larger than 20%), obvious distress after acupuncture treatment so to ensure the their well-being. |
| F. | Possible adverse effects of treatments on animal | | | | YES － please answer the following questions.  NO － please go to Part G |
|  | - | When known, the adverse effects of treatments must be indicated. For novel treatments, provide information on related treatments if known. | | | As for acupuncture for humans, the only anticipated adverse effect resulted from the experiments will be the light pain and sensation feeling when the needles are inserted. |
|  | - | Where serious morbidity is likely, the applicant must stipulate | | |  |
|  |  | - | Frequency of assessment of well-being of animal | | 3 times per week |
|  |  | - | Person(s) who will monitor well-being of animal | | Mr Leung Sin Bond and Dr Lin Zhi-xiu |
|  |  | - | Conditions under which animals will be terminated (e.g. certain % loss of body weight, obvious distress, extent of tumour growth) | | N/A |
| G. | Details of analgesia | | | | N/A |
| H. | Termination – clearly describe method to be used | | | | The animals will be terminated by carbon dioxide suffocation. |
| I. | Justification if non-standard euthanasia method used | | | | N/A |
| J. | Disposal method for carcasses, tissues and contaminated materials | | | | Animal carcasses and associated waste will be disposed according to the "Guideline on Disposing Biological/Clinical Waste in CUHK Campus" provided by University Safety and Environment Office (USEO). |
| K. | Disposal method for live animals | | | | N/A |
