# Supplementary material for: Attenuation of blood pressure in spontaneously hypertensive rats by acupuncture was associated with reduction oxidative stress and improvement from endothelial dysfunction
Source: Chin Med. 2016 Aug 30;11(1):38. doi: 10.1186/s13020-016-0110-0 (PMC5006281; doi:10.1186/s13020-016-0110-0)
Supplement: Supplementary file 4 — 10.1186/s13020-016-0110-0 Standard operating procedure. [file 13020_2016_110_MOESM4_ESM.pdf]

香港中文大學醫學院

Faculty Of Medicine

The Chinese University Of Hong Kong

醫院管理局
新界東醫院聯網
Hospital Authority
New Territories East Cluster

The Joint Chinese University of Hong Kong – New Territories East Cluster Clinical Research Ethics Committee (The Joint CUHK-NTEC CREC)

Standard Operating Procedure

Document Particulars	
Document Type	☒ SOP [(a)] ☐ Guideline [(b)] ☐ Working Manual [(c)]
Document Reference No.	HAHO-REC/SOP/001
Initial Issue Date	7th April, 2014
Version No.	2
Issue Date	3rd March, 2015
Effective Date	3rd March, 2015
Next Review Date	Before 3rd March, 2018

Approval
____<br>Prof. Benny ZEE<br>Chairman<br>The Joint CUHK-NTEC CREC<br><br>Date: 3rd March, 2015

(a) A Standard Operating Procedure (SOP) is an official document outlining the necessary procedures for executing a specified task, which shall be approved by the authorized representative(s) of the organization(s) and complied with by the relevant operating unit(s) and personnel.

(b) A Guideline is a guidance document for elaborating and facilitating compliance with the relevant SOP(s) or requirement(s), which could be approved by the authorized quality assurance specialist(s) and/or the authorized representative(s) of the organization(s).

(c) A Working Manual is a document providing more details about execution of the required procedures under the relevant SOP(s), which could be approved by the authorized representative(s) of the operating unit(s) responsible for the task concerned and followed by the relevant operational personnel.

Blank Page

Version and Review History

Version No.	Issue Date (DD/MM/YY)	Effective Date (DD/MM/YY)	Review Date (DD/MM/YY)	Highlights for the Issue
1			N/A	Enforcement of this new SOP (superseding the previous SOP) for the purpose of enhancing ethics and scientific oversight of clinical studies and harmonizing requirements with other research ethics committees of the Hospital Authority.
2	03/03/15	03/03/15	03/03/15	Revised Section 8.6 Full Review by Phase 1 Panel and minor administration updates throughout the SOP

Blank Page

Table of Contents

Part A: Organization 9

1. Establishment, Mission and Standards 11
   - 1.1 Establishment 11
   - 1.2 Mission 11
   - 1.3 Standards of Establishment and Operations 11
2. Governance 12
   - 2.1 Governance Structure 12
   - 2.2 Powers and Responsibilities of RC and CMC 13
   - 2.3 Collaboration with HAREC 14
3. Jurisdiction, Powers and Responsibilities 14
   - 3.1 Activities under Jurisdiction 14
   - 3.2 Powers and Responsibilities 16
4. Structure and Membership 17
   - 4.1 Organizational Structure 17
   - 4.2 Membership 17
   - 4.3 Chairmanship 19
   - 4.4 Vice/Deputy Chairmanship 20
   - 4.5 Conflicts of Interest of Members 21
   - 4.6 Confidentiality Obligations of Members 22
   - 4.7 Training and Continuous Education for Members 22
5. Compositions and Functions of Review Panels 22
   - 5.1 Review Panels in the IRB/REC 22
   - 5.2 Standard Review Panel 23
   - 5.3 Expedited Review Panel 23
   - 5.4 Phase 1 Clinical Trials Review Panel 24
   - 5.5 SAE Review Panel 25
6. Secretariat 25
   - 6.1 Accountability and Composition of Secretariat 25
   - 6.2 Responsibilities of Secretariat 25
   - 6.3 Confidentiality Obligations of Secretariat Staff 26

6.4 Training and Continuous Education for Secretariat Staff 26
7. Quality Assurance 27
7.1 Standard Operating Procedure, Guidelines and Working Manuals 27
7.2 Audits and Inspections 27
7.3 Registration with U.S. OHRP 28

Part B: Operations 31

8. Initial Review 33
8.1 Initial Review as a Mandatory Requirement 33
8.2 Application for Initial Review 33
8.3 Categorization of Clinical Studies and Assignment of Review Channels 33
8.4 Full Review by Standard Panel 35
8.5 Expedited Review by Expedited Panel 37
8.6 Full Review by Phase 1 Panel 39
9. Continuous Oversight 44
9.1 Importance of Continuous Oversight 44
9.2 Regular Continuing Review 44
9.3 Review of Amendments and Changes 45
9.4 Review of New Information 46
9.5 Review of Deviations and Compliance Incidents 47
9.6 Review of Safety Reports 48
9.7 Final Review 51
10. Study Site Auditing 52
10.1 Purpose and Types of Audits by IRB/REC 52
10.2 Conduct and Follow-up of Audits 52
11. Reevaluation Mechanism 53
11.1 Right to Request for Reevaluation 53
11.2 Reevaluation Process 54
12. Review Fees 54
12.1 Determination of Review Fees 54
12.2 Payment of Review Fees 54
13. Records Management 54
13.1 Central Electronic Database 54

13.2 Records Retention 55

Appendices 57

Appendix 1: List of Defined Terms 59
Appendix 2: Major Premises Covered under this SOP...61
Appendix 3: Organization Chart of The Joint CUHK-NTEC CREC......................................62
Appendix 4: Persons Eligible to Nominate IRB/REC Members..63
Appendix 5: Documents Required for an Application for Initial Review...............................64
Appendix 6: Clinical Study Categorization Form 66
Appendix 7: Common Considerations in IRB/REC Review..69
Appendix 8: Sample Notice for Communicating IRB/REC's Decisions 70
Appendix 9: Safety Events Reporting Requirements 76
Appendix 10: Serious Adverse Events Reporting Process 77

Blank Page

Part A:
Organization

Blank Page

1. Establishment, Mission and Standards

1.1 Establishment

1.1.1 Establishment of IRB/REC: The Joint Chinese University of Hong Kong - New Territores East Cluster Clinical Research Ethics Committee ("**The Joint CUHK-NTEC CREC**", or "**IRB/REC**" in short) was established by The Chinese University of Hong Kong ("**CUHK**") and New Territorries East Cluster ("**NTEC**") in accordance with its terms of reference for overseeing research involving human subjects (hereinafter referred to as "clinical studies") undertaken by and/or conducted in the premises owned, managed and/or controlled by CUHK and/or NTEC, and/or involving patients and/or staff thereof as human subjects in such clinical studies.

1.1.2 Background of CUHK: Founded in 1963, CUHK is a forward-looking comprehensive research university with a global vision and a mission to combine tradition with modernity, and to bring together China and the West. CUHK teachers and students hail from all around the world.

1.1.3 Background of NTEC: NTEC is one of the hospital management clusters under the Hospital Authority (**"HA**"), a statutory body established under the Hospital Authority Ordinance (Chapter 113 of the laws of Hong Kong), and is authorized by the HA to manage the hospitals, clinics and medical facilities within NTEC and to provide medical services therein to the public.

1.2 Mission

1.2.1 IRB/REC's Mission: The mission of the IRB/REC is protecting the rights, safety and well-being of human subjects with respect to their participation in clinical studies through initial review and continuous oversight of such clinical studies from the ethical and scientific perspectives.

1.3 Standards of Establishment and Operations

1.3.1 Core Standards: The IRB/REC is established and operated primarily in compliance with:

(a) the Declaration of Helsinki of the World Medical Association ("**Declaration of Helsinki**");
(b) the International Conference on Harmonization of Technical Requirements for the Registration of Pharmaceuticals for Human Use Guideline for Good Clinical Practice ("**ICH GCP**") (if applicable);

(c) this standard operating procedure ("**SOP**"); and
(d) the guideline(s) and working manual(s) of the IRB/REC (if any).

1.3.2 Standards under Accreditations or Quality Assurance Schemes: The IRB/REC will also observe and comply with the standards as required under the relevant accreditation or quality assurance schemes, such as:

(a) China Good Clinical Practice Guideline for Drug Clinical Trials ("**China GCP**"), as required under the clinical trial organization accreditation by the China Food and Drug Administration ("**CFDA**"); and
(b) U.S. Code of Federal Regulations Title 21 Part 56 ("**21 CFR 56**") about institutional review boards and Title 45 Part 46 ("**45 CFR 46**") about protection of human subjects, as required under the registration with the U.S. Office for Human Research Protections ("**OHRP**").

1.3.3 Other Applicable Standards: In addition, in performing its responsibilities of ethics and scientific review and oversight of clinical studies, the IRB/REC may, as it deems appropriate, take reference of other applicable ethical or scientific principles, such as those set out in:

(a) the Guideline on Ethics Oversight and Scientific Evaluation of Phase 1 Clinical Trials issued by the Consortium on Harmonization of Institutional Requirements for Clinical Research ("**CHAIR Phase 1 Guideline**");
(b) the Hospital Authority Guide on Research Ethics for Study Site & Research Ethics Committee ("**HA Guide**"); and
(c) the Ethical Principles and Guidelines for the Protection of Human Subjects of Research first drafted by the U.S. National Commission for the Protection of Human Subjects of Biomedical and Behavioral Research at the Belmont Conference Center and officially created by the former U.S. Department of Health, Education, and Welfare in 1979 ("**Belmont Report**").

1.3.4 Reference to Other Standards: For the avoidance of doubt, the above is not an exhaustive list of standards and does not prohibit the IRB/REC from complying with or taking reference of other applicable standards.

2. Governance

2.1 Governance Structure

2.1.1 Governance Authority: The IRB/REC is governed by CUHK and NTEC ("**Governing**

Body(ies)"), represented by Research Committee, CUHK and the Cluster Management Committee, NTEC.

2.1.2 Governance Enforcement: Governance of the IRB/REC is enforced through the Research Committee (CUHK) and Cluster Management Committee (NTEC) ("**RC and CMC**") under the authorization of the Governing Body(ies).

2.1.3 Composition of RC and CMC: Composition of RC consists of a CUHK Chairperson and CUHK Faculty representatives and CUHK Senior Academic representatives as members. The CMC consists of a Chairperson (CCE, NTEC/HCE) and CUHK representatives, Hospital representatives and Cluster Administration as members.

2.2 Powers and Responsibilities of RC and CMC

2.2.1 RC and CMC's Responsibilities: The RC and CMC has the responsibilities to:

(a) formulate the policies for ethics and scientific review and oversight of clinical studies (e.g. developing and updating this SOP);
(b) ensure the continuous operation and functioning of the IRB/REC;
(c) oversee compliance of the IRB/REC with this SOP and the relevant standards;
(d) report to the Governing Body(ies) the status of operation of the IRB/REC and any significant issue with respect to the clinical studies under the IRB/REC's oversight; and
(e) perform other duties related to oversight of the IRB/REC and clinical studies as delegated by the Governing Body(ies).

2.2.2 RC and CMC's Powers: The RC and CMC has the powers to:

(a) recommend to the Governing Body(ies) on the governance and management of the IRB/REC (e.g. adjusting the organization structure of the IRB/REC);
(b) recommend to the Governing Body(ies) on allocation of resources (including human, financial and infrastructural resources) to the IRB/REC;
(c) interpret this SOP;
(d) determine and adjust the fees for receipt of applications/submissions and performance of ethics and scientific review and oversight;
(e) access the IRB/REC's records and documents;
(f) audit the IRB/REC's composition, operations, records and facilities; and
(g) exercise other authorities related to oversight of the IRB/REC and clinical studies as delegated by the Governing Body(ies).

2.3 Collaboration with HAREC

2.3.1 HAREC's Mission: For the purposes of upholding the standards of clinical studies, harmonizing research ethics principles and practice, and facilitating communication among the HA's cluster research ethics committees, a Hospital Authority Research Ethics Committee ("**HAREC**") consisting of representatives from the cluster research ethics committees and the HA Head Office ("**HAHO**") was established by the HA. HAREC holds regular and ad hoc meetings to exchange opinions, discuss key issues, and formulate policies and guidelines with respect to ethics and scientific review and oversight of clinical studies in line with its mission.

2.3.2 Collaboration with HAREC: The Governing Body(ies) support(s) the mission of HAREC and is/are willing to, through the RC and CMC and the IRB/REC, join force with HAREC to uphold the standards of clinical studies by:

(a) delegating representative(s) to HAREC;
(b) participating in HAREC meetings; and
(c) contributing to discussion and development of common policies and guidelines.

3. Jurisdiction, Powers and Responsibilities

3.1 Activities under Jurisdiction

3.1.1 IRB/REC's Jurisdiction: The IRB/REC shall be responsible for performing ethics and scientific review and oversight of clinical studies:

(a) undertaken by CUHK and/or NTEC (and/or the employees/appointees/students of CUHK and/or NTEC);
(b) conducted wholly or partially in the premises owned, managed and/or controlled by CUHK and/or NTEC, including (but not limited to) those institutions, hospitals and clinics set out in Appendix 2; and/or
(c) involving the patients and/or employees/appointees/students of CUHK and/or NTEC as human subjects.

For the avoidance of doubt, a clinical study will fall under the IRB/REC's jurisdiction if it fulfills any or all of the above conditions, and involvement of organizations or personnel other than those referred to in Section 3.1.1(a) (e.g. an overseas university or a private practitioner in Hong Kong) in a clinical study or performance of part of a clinical study outside the premises referred to in Section 3.1.1(b) (e.g. recruitment of subjects in the community or performance of imaging assessments in a private

hospital) shall not affect the IRB/REC's jurisdiction over the study. Notwithstanding the above, the IRB/REC's review and approval shall not release a principal investigator from the responsibility of obtaining other necessary approvals for his/her study (e.g. management approval from his/her institution/department, regulatory approval through the Hong Kong Department of Health, or approval by the research ethics committee of a collaborating institution if required).

3.1.2 Definition of Clinical Study: For the purpose of this SOP, a clinical study means any systematic investigation in any medical or scientific discipline with the objective of answering question(s) that may contribute to establishment of theory(ies), principle(s) or generalizable knowledge by processing, analyzing and reporting of information collected from:

(a) human beings (e.g. randomized controlled trial on a medical product or clinical procedure, or observational study following the progression of a disease);
(b) identifiable human materials (e.g. genetic analysis of archived human specimens); and/or
(c) identifiable human data (e.g. medical chart review or case series).

3.1.3 Examples of Medical Products: Medical products may include:

(a) drugs (e.g. chemical drugs, biological drugs and vaccines);
(b) medical devices (e.g. implants, diagnostic kits and imaging machines);
(c) Chinese/herbal medicines (e.g. proprietary/traditional Chinese medicines);
(d) health/nutritional supplements;
(e) cell therapies (e.g. stem cells); and
(f) gene therapies (e.g. viral vectors).

3.1.4 Examples of Clinical Procedures: Clinical procedures may include:

(a) clinical examinations/assessments (e.g. venipuncture);
(b) surgical procedures (e.g. tumor resection);
(c) nursing procedures;
(d) physiotherapies;
(e) occupational therapies;
(f) psychotherapies;
(g) behavioral therapies;
(h) alternative therapies (e.g. acupuncture); and
(i) diagnostic imaging methods (e.g. X-ray examination).

3.1.5 Examples of Activities Not Defined as Clinical Studies: For the avoidance of doubt, clinical studies do not include:

(a) the use of medical products/procedures solely for the purpose of clinical care (e.g. emergency use of an unregistered drug with a patient in a life-threatening condition);
(b) evaluation of individual patients' medical records solely for the purpose of clinical care;
(c) investigation of clinical data for quality assurance purpose (e.g. clinical audits); and
(d) investigation on general statistical information relating to hospital services or disease patterns (e.g. number of hospital admissions per year, year-on-year change in the number of diabetic patients attending a specialist out-patient clinic);

provided that such activities are not intended to form a part of a research project or to derive a research publication.

3.1.6 Discretion to Review Other Research Projects: Notwithstanding the scope defined under this Section 3.1, the IRB/REC shall have the discretion to accept applications for ethics and scientific review of other research projects of a healthcare nature or otherwise (e.g. anonymous health survey or research on anonymised patient data) as it deems appropriate.

3.2 Powers and Responsibilities

3.2.1 IRB/REC's Responsibilities: The IRB/REC has the responsibilities to protect the rights, safety and well-being of human subjects with respect to their participation in clinical studies under its jurisdiction through:

(a) receiving applications for initial review of clinical studies from principal investigators, performing initial ethics and scientific review of such studies, and giving its decision(s)/opinion(s) on each application;
(b) performing continuous ethics and scientific oversight during the period of each approved clinical study and giving its decision(s)/opinion(s);
(c) creating and maintaining necessary records with respect to ethics and scientific review and oversight of clinical studies;
(d) reporting to the RC and CMC the status of operation of the IRB/REC and any significant issue with respect to the clinical studies under the IRB/REC's oversight;
(e) allowing and facilitating audits by the RC and CMC and inspections by

competent regulatory authorities;

(f) promoting the concepts of clinical research ethics; and

(g) perform other duties related to ethics and scientific review and oversight of clinical studies as delegated by the RC and CMC or the Governing Body(ies).

3.2.2 IRB/REC's Powers: The IRB/REC has the powers to:

(a) request for, collect and review information, documents and materials necessary for performance of ethics and scientific review and oversight;

(b) recommend modifications to study designs and arrangements on sound ethical or scientific basis and in line with the IRB/REC's mission;

(c) approve or disapprove clinical studies and give other opinions with respect to the ethical and scientific aspects of such clinical studies;

(d) suspend or terminate any approved clinical study if unacceptable risk to subjects arises;

(e) audit clinical studies to assess compliance with study protocols, the IRB/REC's requirements and other applicable standards and requirements;

(f) disclose information of clinical studies to the RC and CMC, the Governing Body(ies) and competent regulatory authorities; and

(g) exercise other authorities related to ethics and scientific review and oversight of clinical studies as delegated by the RC and CMC or the Governing Body(ies).

4. Structure and Membership

4.1 Organizational Structure

4.1.1 Organizational Components: The IRB/REC consists of:

(a) a chairman ("**Chairman**");

(b) vice/deputy chairmen ("**Vice/Deputy Chairmen**");

(c) review panels; and

(d) a secretariat ("**Secretariat**").

4.1.2 Organization Chart: The IRB/REC's organization chart is set out in Appendix 3.

4.2 Membership

4.2.1 Membership Composition: The IRB/REC shall consist of both genders and with a minimum of five (5) members, including:

(a) at least one (1) member whose primary expertise or area of interest is in medical, clinical or biological sciences or related disciplines (“**Scientific Member**”);
(b) at least one (1) member whose primary expertise or areas of interest is not in medical, clinical or biological sciences or related disciplines (“**Non-scientific Member**”); and
(c) at least one (1) member who is neither directly affiliated with the Governing Body(ies) nor the direct family member of any person directly affiliated with the Governing Body(ies), irrespective of the member’s primary expertise or area of interest (“**Independent Member**”).

For the avoidance of doubt, the roles of Non-scientific Member and Independent Member may be assumed by the same IRB/REC member.

4.2.2 Nomination of Members: The persons eligible to nominate IRB/REC members are listed on Appendix 4. All nominations shall be submitted to the RC and CMC for consideration and recommendation to the Governing Body(ies) for appointment. The RC and CMC shall recommend to the Governing Body(ies) a suitable number of candidates with a suitable mix of backgrounds and expertise as appropriate for supporting the IRB/REC’s responsibilities.

4.2.3 Appointment of Members: The Governing Body(ies) shall consider the candidates recommended by the RC and CMC. All IRB/REC members shall be appointed by the Governing Body(ies) in writing.

4.2.4 Term of Membership: Each term of membership will be up to three (3) years. There is no restriction for reappointment as long as a member continues to fulfill the relevant requirements.

4.2.5 Resignation from Membership: Each member may, at his/her own discretion, resign from the IRB/REC membership any time by notice in writing to the Chairman.

4.2.6 Termination of Membership: Membership of the IRB/REC may be terminated by the Governing Body(ies) anytime in writing if a member no longer fulfills the relevant requirements (e.g. the applicable conditions set out in Sections 4.2.1 and 4.7.1) or is deemed by the Governing Body(ies) unsuitable to continue to be an IRB/REC member.

4.2.7 Members’ Responsibilities: An IRB/REC member has the responsibilities to support accomplishment of the mission and fulfillment of the responsibilities of the IRB/REC by contributing to ethics and scientific review and oversight of clinical studies, such

as:

(a) receiving and reviewing documents and information of clinical studies through the Secretariat;
(b) participating in IRB/REC review meetings;
(c) giving his/her opinions on any application, submission or issue of which he/she participated in review; and
(d) keeping the information of clinical studies he/she reviewed confidential.

4.3 Chairmanship

4.3.1 Appointment of Chairman: The Chairman shall be a member of the IRB/REC and be appointed by the Governing Body(ies).

4.3.2 Term of Chairmanship: Each term of chairmanship will be up to three (3) years. There is no restriction for reappointment as long as the Chairman continues to be an IRB/REC member and fulfill the relevant requirements.

4.3.3 Resignation from Chairmanship: The Chairman may, at his/her own discretion, resign from the chairmanship any time by notice in writing to the Governing Body(ies).

4.3.4 Termination of Chairmanship: Chairmanship of the IRB/REC may be terminated by the Governing Body(ies) anytime in writing if the Chairman no longer fulfills the relevant requirements or is deemed by the Governing Body(ies) unsuitable to continue to be a Chairman.

4.3.5 Chairman's Responsibilities: The Chairman has the responsibilities to support accomplishment of the mission and fulfillment of the responsibilities of the IRB/REC by overseeing the IRB/REC's management and operations, such as:

(a) assigning IRB/REC members to review panels;
(b) managing the Secretariat;
(c) chairing full review meetings (or delegating Vice/Deputy Chairmen to do so on his/her behalf);
(d) reporting to the RC and CMC the status of operation of the IRB/REC and any significant issue with respect to the clinical studies under the IRB/REC's oversight;
(e) facilitating audits by the RC and CMC and inspections by competent regulatory authorities (or delegating Vice/Deputy Chairmen or the Secretariat's staff to do so on his/her behalf);
(f) participating in HAREC meetings (or delegating Vice/Deputy Chairmen or the

Secretariat's staff to do so on his/her behalf); and

(g) performing other duties related to ethics and scientific review and oversight of clinical studies as delegated by the RC and CMC or the Governing Body(ies).

4.3.6 Chairman's Powers: The Chairman has the powers to:

(a) appoint Vice/Deputy Chairmen;
(b) approve this SOP (including its future updates);
(c) develop and approve working manuals, if needed, to facilitate accomplishment of the IRB/REC's responsibilities in line with the principles and requirements of this SOP;
(d) exercise discretion on accepting applications for ethics and scientific review;
(e) initiate audits of clinical studies to assess compliance with study protocols, the IRB/REC's requirements and other applicable standards and requirements;
(f) disclose information of clinical studies to the RC and CMC, the Governing Body(ies) and competent regulatory authorities; and
(g) exercise other authorities related to ethics and scientific review and oversight of clinical studies as delegated by the RC and CMC or the Governing Body(ies).

4.4 Vice/Deputy Chairmanship

4.4.1 Appointment of Vice/Deputy Chairmen: The Chairman may appoint any IRB/REC member as a Vice/Deputy Chairman as he/she deems fit to assist him/her to perform the Chairman's responsibilities. There is no limitation on the number of Vice/Deputy Chairmen.

4.4.2 Term of Vice/Deputy Chairmanship: Each term of vice/deputy chairmanship will be up to three (3) years. There is no restriction for reappointment as long as a Vice/Deputy Chairman continues to be an IRB/REC member and fulfill the relevant requirements.

4.4.3 Resignation from Vice/Deputy Chairmanship: A Vice/Deputy Chairman may, at his/her own discretion, resign from the vice/deputy chairmanship any time by notice in writing to the Chairman.

4.4.4 Termination of Vice/Deputy Chairmanship: Vice/Deputy chairmanship of the IRB/REC may be terminated by the Chairman anytime in writing if the Vice/Deputy Chairman no longer fulfills the relevant requirements or is deemed by the Chairman unsuitable to continue to be a Vice/Deputy Chairman.

4.4.5 Vice/Deputy Chairmen's Responsibilities: A Vice/Deputy Chairman has the responsibilities to support accomplishment of the mission and fulfillment of the responsibilities of the IRB/REC by supporting the Chairman in overseeing the IRB/REC's management and operations, such as:

(a) chairing IRB/REC review meetings as delegated by the Chairman;
(b) facilitating audits by the RC and CMC and inspections by competent regulatory authorities as delegated by the Chairman;
(c) participating in HAREC meetings as delegated by the Chairman; and
(d) performing other duties as delegated by the Chairman to support fulfillment of the Chairman's responsibilities.

4.5 Conflicts of Interest of Members

4.5.1 Avoidance of Conflicts of Interest: Conflicts of interest and potential conflicts of interest may lead to bias in ethics and scientific review and oversight and should be avoided. An IRB/REC member's potential conflicts of interest in a clinical study may include:

(a) any proprietary interest in the study and/or the investigational product(s)/procedure(s) (e.g. patent);
(b) any equity interest in an organization owning the rights to the study and/or the investigational product(s)/procedure(s) (e.g. stocks and options), except for indirect ownership through collective investment schemes (e.g. mutual funds and mandatory provident funds) in which the IRB/REC member has no control over the investment strategy;
(c) any financial payment or valuable provided by an organization owning the rights to the study and/or the investigational product(s)/procedure(s) (e.g. donation);
(d) any financial arrangement linking to the study and/or the investigational product(s)/procedure(s) (e.g. royalty fee);
(e) any decision-making or influential position in an organization owning the rights to the study and/or the investigational product(s)/procedure(s);
(f) a key role in the study team (e.g. principal investigator and co-investigator);
(g) membership to the study's data and safety monitoring committee ("**DSMC**");
(h) leadership to the department/division of any of the study's investigators (e.g. Chief of Service or Head of department/division); and
(i) a direct family relationship with the principal investigator or any key study team member (e.g. spouse).

4.5.2 Declaration of Interest: Each IRB/REC member participating in reviewing a study shall, prior to the review, make a declaration for the absence of any of the interests set out in Section 4.5.1. Any IRB/REC member having any of those interests shall not participate in reviewing the study.

4.6 Confidentiality Obligations of Members

4.6.1 Members' Confidentiality Obligations: All the information disclosed to an IRB/REC member will be deemed confidential and shall not be disclosed to any third party or used for any purpose other than performing the responsibilities of an IRB/REC member, save and except for disclosure to the RC and CMC, the Governing Body(ies) or the relevant regulatory authorities.

4.6.2 Statement of Confidentiality: Upon acceptance of an appointment as an IRB/REC member, the member will be required to sign a statement of confidentiality to confirm his/her agreement to the confidentiality obligations in the IRB/REC.

4.7 Training and Continuous Education for Members

4.7.1 Core Training: IRB/REC members need to acquire knowledge in the core principles of clinical research ethics and the IRB/REC's operations, such as by training on:

(a) the Declaration of Helsinki;
(b) the ICH GCP;
(c) this SOP; and
(d) any applicable guideline or working manual issued by the IRB/REC.

4.7.2 Modes of Training: There is no restriction on the modes of training. Examples of training include participation in workshops/seminars/web-based training programs, sitting for examinations, and self-learning.

4.7.3 Continuous Education: IRB/REC members are also encouraged to receive continuous education in respect of ethics and scientific review and oversight of clinical studies.

4.7.4 Training Records: Any relevant training or continuous education received by an IRB/REC member will need to be documented. The Secretariat will have the responsibility to maintain training records for all IRB/REC members.

5. Compositions and Functions of Review Panels

5.1 Review Panels in the IRB/REC

5.1.1 Existing Review Panels: The IRB/REC's responsibilities of ethics and scientific review and oversight shall be performed by its review panels. The existing review panels include:

(a) a Standard Review Panel ("**Standard Panel**");
(b) an Expedited Review Panel ("**Expedited Panel**");
(c) a Phase 1 Clinical Trials Review Panel ("**Phase 1 Panel**"); and
(d) a Serious Adverse Event (SAE) Review Panel ("**SAE Panel**")

5.1.2 Members' Participation in Review Panels: Each IRB/REC member may be delegated to join one or more review panels.

5.1.3 Adjustment to Review Panels: The RC and CMC may, as it deems appropriate, reorganize the existing review panels, establish new review panels or make adjustments to the review panels' compositions or functions.

5.2 Standard Review Panel

5.2.1 Standard Panel's Responsibility: The Standard Panel is responsible for performing initial ethics and scientific review of clinical studies assigned for initial review through "Channel A" as determined by the clinical study categorization mechanism stipulated in Section 8.3, and continuing review of subsequent applications/submissions that require full review by the Standard Panel as determined by the IRB/REC according to this SOP.

5.2.2 Composition of Standard Panel: The Standard Panel shall consist of both genders and with a minimum of five (5) members, including:

(a) at least one (1) Scientific Member;
(b) at least one (1) Non-scientific Member; and
(c) at least one (1) Independent Member.

For the avoidance of doubt, the roles of Non-scientific Member and Independent Member may be assumed by the same IRB/REC member.

5.2.3 Chairman's Authority to Assign Members to Standard Panel: The Chairman and Vice/Deputy Chairmen shall be members of the Standard Panel. Subject to compliance with the minimum requirements stipulated in Section 5.2.2, the Chairman may assign any number of IRB/REC members to the Standard Panel.

5.3 Expedited Review Panel

5.3.1 Expedited Panel's Responsibility: The Expedited Panel is responsible for performing initial ethics and scientific review of clinical studies assigned for initial review through "Channel B" as determined by the clinical study categorization mechanism stipulated in Section 8.3, and continuing review of subsequent applications/submissions that are eligible for expedited review as determined by the IRB/REC according to this SOP.

5.3.2 Composition of Expedited Panel: The Expedited Panel shall consist of a minimum of two (2) scientific members.

5.3.3 Chairman's Authority to Assign Members to Expedited Panel: Subject to compliance with the minimum requirements stipulated in Section 5.3.2, the Chairman may assign any number of IRB/REC members to the Expedited Panel.

5.4 Phase 1 Clinical Trials Review Panel

5.4.1 Phase 1 Panel's Responsibility: The Phase 1 Panel is responsible for performing initial ethics and scientific review and oversight of phase 1 clinical trials assigned for initial review through "Channel C" as determined by the clinical study categorization mechanism stipulated in Section 8.3, and continuing review of subsequent applications/submissions that require full review by the Phase 1 Panel as determined by the IRB/REC according to this SOP.

5.4.2 Composition of Phase 1 Panel: The Phase 1 Panel shall consist of both genders and with a minimum of five (5) members, including:

(a) at least one (1) Scientific Member;
(b) at least one (1) Non-scientific Member; and
(c) at least one (1) Independent Member.

For the avoidance of doubt, the roles of Non-scientific Member and Independent Member may be assumed by the same IRB/REC member.

5.4.3 Training for Phase 1 Panel Member: Phase 1 Panel members need to acquire extra knowledge in reviewing and overseeing phase 1 clinical trials (in addition to those required under Section 4.7.1), such as by training on the CHAIR Phase 1 Guideline.

5.4.4 Chairman's Authority to Assign Members to Phase 1 Panel: Subject to compliance with the minimum requirements stipulated in Sections 5.4.2 and 5.4.3, the Chairman may assign any number of IRB/REC members to the Phase 1 Panel.

5.5 SAE Review Panel

5.5.1 SAE Panel's Responsibility: The SAE Panel is responsible for timely review and monitoring of serious adverse events and suspected unexpected serious adverse reaction (SUSAR) as reported by the investigator according to the SAE and SUSAR reporting procedure of the IRB/REC.

5.5.2 Composition of SAE Panel: The SAE Panel shall consist of a minimum of two (2) scientific members in each group. The number of groups formed depends on the amount of SAE and SUSAR handled through the reporting mechanism.

5.5.3 Chairman's Authority to Assign Members to Standard Panel: Subject to compliance with the minimum requirements stipulated in Section 5.5.2, the Chairman may assign any number of IRB/REC members to the SAE Panel.

6. Secretariat

6.1 Accountability and Composition of Secretariat

6.1.1 Accountability to Chairman: The Secretariat is directly accountable to the Chairman.

6.1.2 IRB/REC Secretary: The IRB/REC shall designate a staff member of the Secretariat to assume the role of an IRB/REC secretary ("**Secretary**", irrespective of the job title assigned) who shall take charge of the Secretariat's responsibilities and supervise other staff members of the Secretariat.

6.1.3 Secretariat Staff Composition: The Secretariat's staff composition will be determined by the Chairman and the RC and CMC as they deem appropriate.

6.2 Responsibilities of Secretariat

6.2.1 Secretariat's Responsibilities: The Secretariat has the responsibilities to support accomplishment of the mission and fulfillment of the responsibilities of the IRB/REC by providing professional management and administrative support to the IRB/REC and the RC and CMC, such as:

(a) facilitating membership management (e.g. facilitating appointment of IRB/REC members, Chairman and Vice/Deputy Chairmen, and maintaining an updated membership list);
(b) facilitating review, updating and maintenance of this SOP and other relevant guidelines and working manuals;
(c) receiving applications/submissions relating to clinical studies;

(d) facilitating initial review of clinical study applications by the IRB/REC (e.g. setting up review meetings, preparing meeting agendas and minutes, and taking required follow-up actions);
(e) facilitating continuous oversight of clinical studies by the IRB/REC (e.g. collecting and arranging for review of amendments/changes, new information, deviations/compliance incidents, safety reports, progress reports and final reports);
(f) maintaining records of ethics and scientific review and oversight (e.g. initial applications, review meeting minutes and approval letters);
(g) facilitating audits by the RC and CMC and inspections by competent regulatory authorities;
(h) providing administrative support to the IRB/REC and the RC and CMC; and
(i) performing other duties related to ethics and scientific review and oversight of clinical studies as delegated by the Chairman.

6.3 Confidentiality Obligations of Secretariat Staff

6.3.1 Secretariat Staff's Confidentiality Obligations: All the information disclosed to the Secretariat's staff will be deemed confidential and shall not be disclosed to any third party or used for any purpose other than performing the responsibilities of the Secretariat, save and except for disclosure to the RC and CMC, the Governing Body(ies) or the relevant regulatory authorities.

6.3.2 Statement of Confidentiality: Upon acceptance of an appointment as a Secretariat staff member, the staff member will be required to sign a statement of confidentiality to confirm his/her agreement to the confidentiality obligations in the IRB/REC.

6.4 Training and Continuous Education for Secretariat Staff

6.4.1 Core Training: The Secretariat's key staff members need to acquire knowledge in the core principles of clinical research ethics and the IRB/REC's operations, such as by training on:

(a) the Declaration of Helsinki;
(b) the ICH GCP;
(c) this SOP; and
(d) any applicable guideline or working manual issued by the IRB/REC.

6.4.2 Modes of Training: There is no restriction on the modes of training. Examples of training include participation in workshops/seminars/web-based training programs,

sitting for examinations, and self-learning.

6.4.3 Continuous Education: The Secretariat's staff members are also encouraged to receive continuous education in respect of ethics and scientific review and oversight of clinical studies.

6.4.4 Training Records: Any relevant training or continuous education received by a staff member of the Secretariat will need to be documented. The Secretariat will have the responsibility to maintain training records for all its staff members.

7. Quality Assurance

7.1 Standard Operating Procedure, Guidelines and Working Manuals

7.1.1 Approval of SOP: This SOP is approved by the Chairman. The originally signed copy shall be kept by the Secretariat.

7.1.2 Review of SOP: This SOP will be reviewed by reviewer(s) delegated by the Chairman or the RC and CMC at least every three (3) years. Additional reviews may be performed as deemed required by the Chairman or the RC and CMC.

7.1.3 Updating of SOP: The Chairman and the RC and CMC will duly consider the recommendations by the reviewer(s) in order to finalize an updated SOP. Whether or not any change is made to the SOP,

(a) the version and review history at the front part of the SOP shall be updated;
(b) the updated SOP shall be approved by the Chairman by signing on the cover page; and
(c) the originally signed copy shall be kept by the Secretariat.

7.1.4 Guidelines and Working Manuals: The IRB/REC may, as it deems required, develop and maintain guidelines and/or working manuals to supplement this SOP. The Chairman shall have the authority to approve guidelines and working manuals. In the event of any conflict or inconsistency between a guideline/working manual and this SOP, this SOP shall prevail.

7.2 Audits and Inspections

7.2.1 Responsibility to Facilitate Audits/Inspections: The IRB/REC will allow and facilitate audits by the RC and CMC and inspections by competent regulatory authorities on the IRB/REC's composition, operations, records and facilities on reasonable request. An

audit/inspection will be performed by auditor(s) delegated by the RC and CMC or by inspector(s) delegated by the competent regulatory authority.

7.2.2 Preparation for Audits/Inspections: Any request for audit/inspection shall be made to the Chairman. Upon receipt of a request, the IRB/REC will:

(a) verify the legitimacy of the request;
(b) designate a person to take charge of the audit/inspection;
(c) liaise with the auditing/inspection body on the scope, schedule and arrangements for the audit/inspection;
(d) make all necessary documents, records and materials available for the audit/inspection; and
(e) do other preparation as needed.

7.2.3 Facilitation of Audits/Inspections: During an audit/inspection, the IRB/REC will:

(a) confirm the identity(ies) of the auditor(s)/inspector(s) at the start of the audit/inspection;
(b) cooperate with the auditor(s)/inspector(s) to facilitate a smooth audit/inspection; and
(c) monitor the auditing/inspection process and record any significant issue or finding from the audit/inspection.

7.2.4 Follow-up on Audits/Inspections: After completion of an audit/inspection, the IRB/REC will:

(a) collect a written audit/inspection report from the auditor(s)/inspector(s);
(b) respond to the auditing/inspection body on any issue or finding highlighted in the audit/inspection report;
(c) take proper follow-up action(s) with respect to each issue or finding;
(d) issue a follow-up report to the auditing/inspection body upon completion of all follow-up action(s) if so required by the auditing/inspection body; and
(e) keep a complete record for the audit/inspection.

7.3 Registration with U.S. OHRP

7.3.1 Registration with OHRP: As required under U.S. regulations, any organization that wishes to be involved in any clinical study funded by the U.S. federal government or any U.S. governmental agencies (e.g. the U.S. National Institutes of Health ("**NIH**")) must use ethics committee(s)/institutional review board(s) registered with the OHRP

for review and oversight of its clinical studies. To qualify the Governing Body(ies) to participate in such U.S.-funded clinical studies, the IRB/REC has registered with the OHRP.

7.3.2 Compliance with OHRP Requirements: Under the requirement of the registration with the OHRP, the IRB/REC will need to observe and comply with the applicable requirements for registration, including:

(a) 21 CFR 56 about institutional review boards; and
(b) 45 CFR 46 about protection of human subjects.

7.3.3 Maintenance of Registration: The IRB/REC will need to continuously maintain a valid registration, in particular:

(a) a registration must be renewed every three (3) years; and
(b) any change of registration information regarding the IRB/REC's Chairman or contact person (e.g. the Secretary) must be updated within ninety (90) days after the change occurs.

Blank Page

Part B:
Operations

Blank Page

8. Initial Review

8.1 Initial Review as a Mandatory Requirement

8.1.1 Objective of Initial Review: An initial IRB/REC review is the ethics and scientific review by the IRB/REC prior to initiation of a proposed clinical study. The objective is to evaluate the ethical and scientific aspects of a proposed clinical study in order to protect the rights, safety and well-being of human subjects who may or will participate in the study.

8.1.2 Requirement for Prior Approval: The IRB/REC's initial review and prior written approval is a mandatory requirement for initiation of any clinical study under the IRB/REC's jurisdiction as stipulated in Section3.1.

8.2 Application for Initial Review

8.2.1 Principal Investigators as Applicants: Submitting an application to the IRB/REC for initial review of a clinical study is the responsibility of the study's principal investigator (who shall act as the applicant under the application). For the purpose of an application, the principal investigator of a study is the investigator who takes the final responsibility for the conduct of the study at his/her study site and shall be an employee/appointee/student of the Governing Body(ies) (irrespective of any other title assigned to him/her in the study). In the event of a multicentre study, the investigator who takes responsibility for the entire multicentre study may be referred to as the lead investigator (who may or may not be the same as the principal investigator).

8.2.2 Submission of Applications: All applications shall be submitted through the Secretariat. Principal investigators are required to observe the review meeting schedule and application submission deadlines announced by the IRB/REC from time to time for the purpose of time planning, and shall comply with the IRB/REC's requirements in compiling and submitting their applications.

8.2.3 Application Documents: Each application shall include (but not limited to) the documents required as listed on Appendix 5. The IRB/REC may request for additional documents, information or clarification as it reasonably deems required, and has the right to refuse performing an initial review if an application is incomplete and/or insufficient information is made available to the IRB/REC.

8.3 Categorization of Clinical Studies and Assignment of Review Channels

8.3.1 Principles of Study Categorization: To enhance the efficiency and effectiveness of initial reviews, the IRB/REC adopts a risk categorization approach by categorizing clinical studies based on six groups of risk factors including:

(a) involvement of human subject recruitment;
(b) subject vulnerability;
(c) subject assignment methods;
(d) involvement of medical products;
(e) involvement of clinical procedures; and
(f) study designs.

8.3.2 Mechanism for Study Categorization and Review Channels: The detailed mechanism for categorization of clinical studies is set out on the "Clinical Study Categorization Form" set out in Appendix 6. Principal investigators are required to complete and submit the form together with each application for initial review. Upon receipt of an application, the Secretariat will verify the information on the form and arrange for initial review through one of the following review channels:

(a) Channel A: Full review by the Standard Panel
(b) Channel B: Expedited review by the Expedited Panel
(c) Channel C: Full review by the Phase 1 Panel / Recommend the principal investigator to collaborate with a study site under the jurisdiction of a research ethics committee with a specific review panel for phase 1 clinical trials

8.3.3 Chairman's Authority to Assign Review Channel: Notwithstanding the result of categorization under the aforesaid mechanism, the Chairman or Vice/Deputy Chairman (or designee) shall have the authority to:

(a) re-assign an application for expedited review if the study is a multicentre study which has already been approved by any research ethics committee under the HA, and no substantial difference is anticipated with respect to protection of the rights, safety and well-being of subjects whether the study is conducted by the applying principal investigator or by another approved principal investigator; or
(b) re-assign an application for review through any of the other channels at his/her reasonable discretion.

8.3.4 Continuing Review through the Review Channels: Unless otherwise specified in this SOP, continuing review of submissions for approved clinical studies will also be performed through the aforesaid review channels in accordance with the requirements

detailed in Section 9.

8.4 Full Review by Standard Panel

8.4.1 Meeting Schedule: The Standard Panel shall perform full review of applications/submissions by holding regular review meetings at a frequency as the IRB/REC determines, and ad hoc review meetings as the IRB/REC deems necessary. The Secretariat will use its endeavors to work out and make accessible to the investigators an updated meeting schedule for the regular review meetings, together with the submission deadlines corresponding to the meetings, at least for the two (2) subsequent meetings at any point of time to facilitate time planning by investigators for their upcoming studies.

8.4.2 Quorum and Composition of Reviewers: The quorum for a Standard Panel review meeting is five (5) and the composition of the reviewers participating in a review meeting shall fulfill the minimum requirements as stipulated in Section 5.2.2. Each review meeting will be chaired by the Chairman or a Vice/Deputy Chairman. The Secretariat will be responsible for inviting Standard Panel members to participate as reviewers in each review meeting.

8.4.3 Expert Advisors: The Chairman or Vice/Deputy Chairman may, as he/she deems beneficial to the review of an application/submission, invite expert advisor(s) to participate in a review meeting or provide expert advice on an application/submission, provided that each expert advisor shall sign a statement of confidentiality. The expert advisor(s) shall not be eligible to vote for the application/submission.

8.4.4 Pre-meeting Review: For each application/submission assigned for full review by the Standard Panel, the Secretariat will, prior to the review meeting, send the application/submission (together with all the relevant documents) to the reviewers at least seven (7) calendar days before the review meeting for performing pre-meeting review. The Secretariat may, at its discretion, forward the reviewers' preliminary opinions, if any, to the principal investigator for consideration before the review meeting.

8.4.5 Investigator's Participation in a Meeting: The Chairman or Vice/Deputy Chairman may, as he/she deems beneficial to the review of an application/submission, request a principal investigator (or his/her designee) to participate and/or present the application/submission in a review meeting.

8.4.6 Conduct of Meeting: The Chairman or Vice/Deputy Chairman will use his/her endeavors to facilitate a balanced discussion among the participating reviewers in

order to reach an ethically and scientifically satisfactory decision on each application/submission.

8.4.7 Scope of Considerations: In performing a review, the reviewers will evaluate and discuss the ethical and scientific aspects of the study for the purpose of protecting the rights, safety and well-being of human subjects, and in particular from six key dimensions including:

(a) research products/procedures;
(b) study design;
(c) study execution;
(d) subjects' rights;
(e) potential research biases; and
(f) potential liability management.

A list of common considerations corresponding to the six key dimensions is set out in Appendix 7. For the avoidance of doubt, the said list and the selected items are only provided for reference but should not be taken as an exhaustive checklist for performing a review.

8.4.8 Decision by Consensus: The Chairman or Vice/Deputy Chairman will use his/her endeavors to facilitate the panel's decision on each application/submission by thorough discussion and unanimous consensus.

8.4.9 Decision by Voting: In the event that a unanimous consensus on an application/submission cannot be reached within a reasonable period of discussion, the Chairman or Vice/Deputy Chairman may at his/her discretion call for resolution by voting. A reviewer may vote for or against an application/submission, or otherwise abstain from voting. A resolution shall be approved by majority vote of at least 75% of the reviewers who are eligible to vote for the application/submission. Any reviewer who has a conflict of interest or potential conflict of interest in an application/submission shall make a declaration and is not eligible to vote. The reviewer(s) dissenting and/or abstaining (together with the reason(s) for dissenting/abstaining) should be recorded in the minutes.

8.4.10 Types of Decisions: After reviewing an application/submission, the review panel will:

(a) approve the application/submission, if it is deemed fulfilling all the relevant requirements of the IRB/REC;
(b) disapprove the application/submission, if any fundamental inconsistency exists

between the application/submission and the IRB/REC's requirements, and such inconsistency is deemed non-rectifiable;

(c) request the principal investigator to modify the application/submission or to provide clarification or further information about the application/submission; or

(d) give other opinion(s) or take other action(s) as it reasonably determines.

8.4.11 Resolution of Queries: In the event that a request for modification, clarification or further information is made by the review panel, the Secretariat will, as soon as possible and within ten (10) working days from the date of a review meeting, send the request to the principal investigator. The principal investigator is required to feedback on the request in writing as soon as possible. The request will be deemed satisfactorily addressed by the principal investigator if no further comment/query is made by the reviewers within a reasonable period as determined by the Secretariat. In the event that the principal investigator does not feedback on the request within ninety (90) days from the date of the request, the review may be terminated by the IRB/REC. In the event that the request is not deemed by all the reviewers satisfactorily addressed by the principal investigator, further queries may be made to the principal investigator, or the application/submission may be brought up for discussion in another review meeting.

8.4.12 Notification of Decisions: The decision on an application/submission will be notified to the principal investigator by the Chairman or Vice/Deputy Chairman (or designee) in writing as soon as possible and within ten (10) working days after the decision is made. A sample notice for communicating decisions to principal investigators is set out in Appendix 8 for reference.

8.4.13 Documentation of Review: The Secretariat will be responsible for documenting and maintaining records for review of each application/submission, such as:

(a) review meeting agenda;

(b) review meeting minutes;

(c) list of reviewers;

(d) each reviewer's conflicts of interest declaration; and

(e) the decision in writing (e.g. letter of approval).

8.5 Expedited Review by Expedited Panel

8.5.1 Review Schedule: Expedited review of an application/submission shall be performed by reviewer(s) in the Expedited Panel upon receipt of the application/submission by the Secretariat and assignment of the application/submission for expedited review by

the IRB/REC as per Section 8.3.

8.5.2 Assignment of Reviewer(s): For each application/submission assigned for expedited review, the Secretariat will send the application/submission (together with all the relevant documents) to at least two (2) reviewers in the Expedited Panel for review.

8.5.3 Scope of Considerations: In performing an expedited review, the reviewer(s) will evaluate the study for the purpose of protecting the rights, safety and well-being of human subjects by taking into account the same ethical and scientific considerations as in a full review, and in particular the common considerations set out in Appendix 7.

8.5.4 Decision by Consensus: The reviewers will use their endeavors to reach a decision on the application/submission by unanimous consensus. A decision by an expedited review may be tabled or endorsed in a full review meeting as the IRB/REC deems required.

8.5.5 Types of Decisions: After reviewing an application/submission, the reviewer(s) will:

(a) approve the application/submission, if it is deemed fulfilling all the relevant requirements of the IRB/REC;
(b) request the principal investigator to modify the application/submission or to provide clarification or further information about the application/submission;
(c) channel the application/submission for full review, if the reviewer(s) has/have a negative opinion on the application/submission and deem(s) a full review is needed; or
(d) give other opinion(s) or take other action(s) as the reviewer(s) reasonably determine(s).

In no circumstances an application/submission can be disapproved only through an expedited review.

8.5.6 Resolution of Queries: In the event that a request for modification, clarification or further information is made by the reviewer(s), the Secretariat will, within ten (10) working days from the date of the request, send the request to the principal investigator. The principal investigator is required to feedback on the request in writing as soon as possible. The request will be deemed satisfactorily addressed by the principal investigator if no further comment/query is made by the reviewer(s) within a reasonable period as determined by the Secretariat. In the event that the principal investigator does not feedback on the request within ninety (90) days from the date of the request, the review may be terminated by the IRB/REC. In the event that the

request is not deemed by all the reviewer(s) satisfactorily addressed by the principal investigator, further queries may be made to the principal investigator, or the application/submission may be channeled for full review.

8.5.7 Notification of Decisions: The decision on an application/submission will be notified to the principal investigator by the Chairman or Vice/Deputy Chairman (or designee) in writing as soon as possible and within ten (10) working days after the decision is made. A sample notice for communicating decisions to principal investigators is set out in Appendix 8 for reference.

8.5.8 Documentation of Review: The Secretariat will be responsible for documenting and maintaining records for review of each application/submission, such as:

(a) the list of reviewer(s);
(b) each reviewer's conflicts of interest declarations; and
(c) the decision in writing (e.g. letter of approval).

8.6 Full Review by Phase 1 Panel

8.6.1 Responsibility of Phase 1 Panel: The Phase 1 Panel dedicates to undertake ethics and scientific review and oversight of phase 1 clinical trials under the IRB/REC's jurisdiction. For the purpose of this SOP, a phase 1 clinical trial means a clinical trial on a new chemical or biological drug not registered in Hong Kong (i.e. investigational medicinal product ("**IMP**")) and fulfills any of the following criteria:

(a) A clinical trial which is designated a phase 1 clinical trial on its protocol.
(b) A clinical trial on an IMP which is tested in humans for the first time.
(c) A clinical trial with only human pharmacology, toxicity and/or safety (but not efficacy) of an IMP as its primary objective(s).
(d) A clinical trial which is reasonably deemed by the IRB/REC a phase 1 clinical trial or equivalent to a phase 1 clinical trial from the perspective of clinical risk.

8.6.2 Review Schedule: Review of an application/submission for a phase 1 clinical trial will be performed upon receipt of the application/submission by the Secretariat and assignment of the application/submission for full review by the Phase 1 Panel as per Section 8.3.

8.6.3 Components of Full Review by Phase 1 Panel: A full review by the Phase 1 Panel consists of two parts, including:

(a) ethics review (which is required for all initial and continuing

applications/submissions for phase 1 clinical trials); and

(b) scientific review (which is applicable to all initial applications and continuing applications/submissions for phase 1 clinical trials if deemed required by the IRB/REC).

8.6.4 Scientific Review by SRP: Scientific review will be performed, if deemed required by the IRB/REC, by a Scientific Review Panel ("**SRP**") to be formed under the Joint Scientific Committee for Phase 1 Clinical Trials ("**JSC**") established and operated by the Consortium on Harmonization of Institutional Requirements for Clinical Research ("**CHAIR**"). For the avoidance of doubt, the SRP only provides its recommendations to the Phase 1 Panel on the scientific aspects of a clinical study but does not itself approve or disapprove an application/submission.

8.6.5 Formation of SRP: If deemed required by the IRB/REC, for each application/submission assigned for full review by the Phase 1 Panel, the Secretariat will, on behalf of the Phase 1 Panel, invite any one (1) member from the JSC to be the lead scientific reviewer, provided that the invited lead scientific reviewer shall not be an employee of the principal investigator's employing institution. The IRB/REC will then recommend to the lead scientific reviewer a minimum of two (2) other members from the JSC (who may or may not be employees of the principal investigator's employing institution) as scientific reviewers to form a SRP for the study. In the event that the IRB/REC is unable to identify sufficient number of suitable and available scientific reviewers from the JSC, the IRB/REC may recommend suitable candidate(s) to CHAIR for consideration for appointment as JSC member(s). Any JSC member having a conflict of interest or potential conflict of interest in the study shall not accept the Phase 1 Panel's invitation as a scientific reviewer and shall not participate in the scientific review. To facilitate an invited JSC member to assess if he/she has a conflict of interest or potential conflict of interest in the study, the Secretariat will provide basic information about the study including at least:

(a) the name of the study's sponsor(s) or coordinating organization(s);
(b) the name or identifying code of the investigational product; and
(c) the name of the principal investigator and other key study team members.

8.6.6 Transfer of Documents to SRP: Upon formation of a SRP, if deemed required by the IRB/REC, the Secretariat will provide to each scientific reviewer a scientific review package (by courier, electronic mail, facsimile or otherwise) including the relevant scientific information in the application/submission, such as:

(a) the completed clinical research ethics review application form;
(b) the study protocol;
(c) the investigator's brochure and/or other documents detailing the nature, properties, pre-clinical data and, if available, human data about the investigational product;
(d) the principal investigator's updated curriculum vitae; and
(e) a scientific reviewer's conflicts of interest declaration form.

8.6.7 Conduct of Scientific Review: If deemed required by the IRB/REC, the SRP will perform scientific review of an application/submission based on the JSC's SOP and the relevant parts of the CHAIR Phase 1 Guideline. The SRP may, as it deems beneficial to the scientific review, request for a meeting with the principal investigator (or his/her designee).

8.6.8 Recommendations by SRP: If deemed required by the IRB/REC, the Secretariat will use its endeavors to solicit the SRP's recommendations (or otherwise the SRP's request for additional information or clarification) through the lead scientific reviewer within ten (10) working days from the day of dispatch of the scientific review package to the scientific reviewers. In the event that the SRP requests for additional information or clarification, the Secretariat will use its endeavors to collect and provide such additional information or clarification to the SRP as soon as reasonably practicable, and further follow up with the SRP within ten (10) working days from the day of dispatch of such additional information or clarification to the scientific reviewers.

8.6.9 Ethics Review by the Phase 1 Panel: Ethics review will be performed, in parallel of a scientific review, through a Phase 1 Panel review meeting.

8.6.10 Quorum and Composition of Reviewers: The quorum for a Phase 1 Panel review meeting is five (5) and the composition of the reviewers participating in a review meeting shall fulfill the minimum requirements as stipulated in Section 5.4.2. Each review meeting will be chaired by the Chairman or a Vice/Deputy Chairman (who is appointed as a member of the Phase 1 Panel). The Secretariat will be responsible for inviting Phase 1 Panel members to participate as reviewers in each review meeting.

8.6.11 Expert Advisors: The Chairman or Vice/Deputy Chairman may, as he/she deems beneficial to the review of an application/submission, invite expert advisor(s) to participate in a review meeting or provide expert advice on an application/submission, provided that each expert advisor shall sign a statement of confidentiality. The expert advisor(s) shall not be eligible to vote for the application/submission.

8.6.12 Pre-meeting Review: For each application/submission assigned for full review by the Phase 1 Panel, the Secretariat will, prior to the review meeting, send the application/submission (together with all the relevant documents) to the reviewers at least seven (7) calendar days before the review meeting for performing pre-meeting review. The Secretariat may, at its discretion, forward the reviewers' preliminary opinions, if any, to the principal investigator for consideration before the review meeting.

8.6.13 Investigator's Participation in a Meeting: The Chairman or Vice/Deputy Chairman may, as he/she deems beneficial to the review of an application/submission, request a principal investigator (or his/her designee) to participate and/or present the application/submission in a review meeting.

8.6.14 Conduct of Meeting: The Chairman or Vice/Deputy Chairman will use his/her endeavors to facilitate a balanced discussion among the participating reviewers in order to reach an ethically and scientifically satisfactory decision on each application/submission.

8.6.15 Scope of Considerations: In performing a review, the reviewers will:

(a) evaluate and discuss the study's ethical aspects in accordance with the relevant parts of the CHAIR Phase 1 Guideline and the common ethical considerations set out in Appendix 7; and
(b) consider the SRP's recommendations with respect to the study's scientific aspects, if applicable.

8.6.16 Decision by Consensus: The Chairman or Vice/Deputy Chairman will use his/her endeavors to facilitate the panel's decision on each application/submission by thorough discussion and unanimous consensus.

8.6.17 Decision by Voting: In the event that a unanimous consensus on an application/submission cannot be reached within a reasonable period of discussion, the Chairman or Vice/Deputy Chairman may at his/her discretion call for resolution by voting. A reviewer may vote for or against an application/submission, or otherwise abstain from voting. A resolution shall be approved by majority vote of at least 75% of the reviewers who are eligible to vote for the application/submission. Any reviewer who has a conflict of interest or potential conflict of interest in an application/submission shall make a declaration and is not eligible to vote. The reviewer(s) dissenting and/or abstaining (together with the reason(s) for dissenting/abstaining) should be recorded in the minutes.

8.6.18 Types of Decisions: After reviewing an application/submission, the review panel will:

(a) approve the application/submission, if it is deemed fulfilling all the relevant requirements of the IRB/REC;
(b) disapprove the application/submission, if any fundamental inconsistency exists between the application/submission and the IRB/REC's requirements, and such inconsistency is deemed non-rectifiable;
(c) request the principal investigator to modify the application/submission or to provide clarification or further information about the application/submission; or
(d) give other opinion(s) or take other action(s) as it reasonably determines.

8.6.19 Resolution of Queries: In the event that a request for modification, clarification or further information is made by the review panel, the Secretariat will, as soon as possible and within ten (10) working days from the date of a review meeting, send the request to the principal investigator. The principal investigator is required to feedback on the request in writing as soon as possible. The request will be deemed satisfactorily addressed by the principal investigator if no further comment/query is made by the reviewers within a reasonable period as determined by the Secretariat. In the event that the principal investigator does not feedback on the request within ninety (90) days from the date of the request, the review may be terminated by the IRB/REC. In the event that the request is not deemed by all the reviewers satisfactorily addressed by the principal investigator, further queries may be made to the principal investigator, or the application/submission may be brought up for discussion in another review meeting.

8.6.20 Notification of Decisions: The decision on an application/submission will be notified to the principal investigator by the Chairman or Vice/Deputy Chairman (or designee) in writing as soon as possible and within ten (10) working days after the decision is made. A sample notice for communicating decisions to principal investigators is set out in Appendix 8 for reference.

8.6.21 Documentation of Review: The Secretariat will be responsible for documenting and maintaining records for review of each application/submission, such as:

(a) review meeting agenda;
(b) review meeting minutes;
(c) list of reviewers (including both the reviewers in the Phase 1 Panel and scientific reviewers in the SRP, if applicable);
(d) each reviewer's conflicts of interest declaration;
(e) the SRP's recommendations, if applicable; and

(f) the decision in writing (e.g. letter of approval).

9. Continuous Oversight

9.1 Importance of Continuous Oversight

9.1.1 Objective of Continuous Oversight: In addition to an initial review, the IRB/REC has the responsibility to continuously oversee the status of each approved and ongoing clinical study for the purpose of continuously protecting the rights, safety and well-being of human subjects participating in the study.

9.1.2 Modes of Continuous Oversight: The IRB/REC will perform continuous oversight of each approved clinical study, until its completion or early termination, by:

(a) regular continuing review;
(b) review of amendments and changes;
(c) review of new information;
(d) review of deviations and compliance incidents;
(e) review of safety reports; and
(f) final review.

9.2 Regular Continuing Review

9.2.1 Frequency of Regular Continuing Review: The IRB/REC shall keep track of the updated status of each approved clinical study through regular continuing review once a year from the date of the initial approval and during the period of the study, or more frequently if deemed required by the IRB/REC considering the degree of risk of a study.

9.2.2 Progress Report: To facilitate the IRB/REC's continuing review, a principal investigator shall have the responsibility to submit a progress report on his/her study to the IRB/REC within one (1) month prior to each deadline for regular continuing review by using the IRB/REC's specified form. The progress report shall include updated study information with respect to the period of review, such as:

(a) the status of the study (e.g. ongoing);
(b) the numbers of subjects recruited in, withdrew from and completed the study;
(c) summary of major changes to the study;
(d) summary of serious adverse events;
(e) summary of complaints by subjects; and

(f) summary of significant updated information that may affect the safety of subjects or subjects' willingness to continue participating in the study.

9.2.3 Review of Progress Reports: Each progress report will be reviewed by reviewer(s) in the Expedited Panel through an expedited review process as stipulated in Section 8.5. In the event that the reviewer(s) deem(s) any information in a progress report may be linked with a substantially higher degree of risk and a full review is required, the submission will be channeled for full review. In no circumstance a study can be terminated only by expedited review.

9.2.4 Notification of Decisions: The decision on a submission will be notified to the principal investigator by the Chairman or a Vice/Deputy Chairman (or designee) in writing as soon as possible and within ten (10) working days after the decision is made. A sample notice for communicating decisions to principal investigators is set out in Appendix 8 for reference.

9.2.5 Reminder by Secretariat: The Secretariat will send a reminder to the principal investigator about one (1) to two (2) months prior to each deadline for regular continuing review. Notwithstanding the above, principal investigators shall anyhow have the responsibility to submit progress reports to the IRB/REC whether or not reminders are received from the Secretariat.

9.2.6 Failure to Submit Progress Report: In the event that a principal investigator fails to submit a progress report to the IRB/REC by the deadline for regular continuing review, the IRB/REC may:

(a) request for suspension of all subject recruitment activities and recruitment of additional subjects into the study;
(b) refuse accepting any new application for initial review of clinical study submitted by the principal investigator and his/her participation in any new clinical study (whether as principal investigator, co-investigator/sub-investigator or otherwise); and/or
(c) notify the issue to the other research ethics committees under the HA, which may refuse his/her participation in any new clinical study (whether as principal investigator, co-investigator/sub-investigator or otherwise);

until the progress report is properly submitted and an approval is received from the IRB/REC.

9.3 Review of Amendments and Changes

9.3.1 Implementation of Amendments/Changes: Investigators and study personnel have the responsibility to adhere to the study protocol and other study documents/materials approved by the IRB/REC. No amendment or change to any approved study document/material shall be implemented without the IRB/REC's approval, except:

(a) where necessary to eliminate any immediate hazard to the subjects; or
(b) if an amendment/change is only of an administrative or logistical nature (e.g. correction of typo errors).

9.3.2 Application for Amendments/Changes: In the event that any amendment or change needs to be made to any study document/material, the principal investigator shall submit an application for study amendment(s)/change(s) to the IRB/REC by using the IRB/REC's specified form.

9.3.3 Review of Amendments/Changes: The Chairman or a Vice/Deputy Chairman (or designee) will perform a preliminary review of an application for study amendment(s)/change(s) and assess the possible change in the degree of risk arising from the proposed amendment(s)/change(s). An application for amendment(s)/change(s) that is/are deemed adding no more than minimal additional risk to the subjects will be reviewed by reviewer(s) in the Expedited Panel through an expedited review process as stipulated in Section 8.5. In the event that the Chairman or Vice/Deputy Chairman (or designee) deems the proposed amendment(s)/change(s) may incur more than minimal additional risk and a full review is required, the application will be channeled for full review. In no circumstance a study can be terminated only by expedited review.

9.3.4 Notification of Decisions: The decision on an application will be notified to the principal investigator by the Chairman or Vice/Deputy Chairman (or designee) in writing as soon as possible and within ten (10) working days after the decision is made. A sample notice for communicating decisions to principal investigators is set out in Appendix 8 for reference.

9.4 Review of New Information

9.4.1 Reporting of New Information: A principal investigator has the responsibility to report to the IRB/REC, by using the IRB/REC's specified form, any new information that may adversely affect the rights, safety or well-being of the subjects or the proper conduct of his/her clinical study.

9.4.2 Review of New Information: The Chairman or a Vice/Deputy Chairman (or designee)

will perform a preliminary review of the new information received and assess if such information may change the risk assessment for the study. If the new information is not deemed to substantially and adversely affect the subjects' rights, safety or well-being, the submission will be reviewed by reviewer(s) in the Expedited Panel through an expedited review process as stipulated in Section 8.5. In the event that the Chairman or Vice/Deputy Chairman (or designee) deems the new information may be linked with a substantially higher degree of risk and a full review is required, the submission will be channeled for full review. In no circumstance a study can be terminated only by expedited review.

9.4.3 Notification of Decisions: The decision on a submission will be notified to the principal investigator by the Chairman or Vice/Deputy Chairman (or designee) in writing as soon as possible and within ten (10) working days after the decision is made. A sample notice for communicating decisions to principal investigators is set out in Appendix 8 for reference.

9.5 Review of Deviations and Compliance Incidents

9.5.1 Reporting of Deviations/Incidents: A principal investigator has the responsibility to report to the IRB/REC, by using the IRB/REC's specified form, any deviation from the study protocol or compliance incident that has occurred during a study and may adversely affect the rights, safety or well-being of any subject, within thirty (30) calendar days from the first awareness of the deviation/incident by the principal investigator.

9.5.2 Review of Reports on Deviations/Incidents: The Chairman or a Vice/Deputy Chairman (or designee) will perform a preliminary review of a report on a deviation/incident and assess if a full review or expedited review is required. If a reported deviation/incident is not deemed to have a substantial adverse effect to the rights, safety or well-being of any subject and no special action will need to be taken by the IRB/REC, the submission will be reviewed by reviewer(s) in the Expedited Panel through an expedited review process as stipulated in Section 8.5. In the event that the Chairman or Vice/Deputy Chairman deems the deviation/incident may result in any substantial adverse effect to the rights, safety or well-being of any subject and special action(s) may need to be taken by the IRB/REC, the submission will be channeled for full review. In no circumstance a study can be terminated only by expedited review.

9.5.3 Notification of Decisions: The decision on a submission will be notified to the principal investigator by the Chairman or Vice/Deputy Chairman (or designee) in

writing as soon as possible and within ten (10) working days after the decision is made. In cases where there is no concern or comment on the deviations/incidents, an acknowledgement of receipt of the submission will be issued to the principal investigator.

9.5.4 Rectification/Remedial/Modification Actions: The IRB/REC will have the right to:

(a) request the principal investigator to take appropriate rectification, remedial and/or modification action(s) with respect to the deviation/incident;
(b) request for suspension of further recruitment of subjects into the study until the required rectification/remedial/modification action(s) has/have been completed; and/or
(c) request for suspension or termination of the study if the required rectification/remedial/modification action(s) is/are not completed within a reasonable period of time, or if the deviation/incident is deemed by the IRB/REC seriously affecting the rights, safety or well-being of the subjects and the deviation/incident is not rectifiable/remediable/modifiable.

9.6 Review of Safety Reports

9.6.1 Safety Monitoring: Continuous safety monitoring is an important part in subject protection in clinical studies. An investigator has the responsibility to:

(a) monitor his/her subjects' safety by observing any safety event occurred in any of the subjects; and
(b) in the event of a multicentre clinical study, observe any significant safety event reported from any other study site.

9.6.2 Types of Safety Events: Considering the severity, foresee ability and causality with an investigational product/procedure, a safety event can be classified as:

(a) an adverse event ("**AE**"), which is an unfavorable or unintended sign, symptom, reaction or disease that is associated in time with participation in a clinical study or the use of an investigational product/procedure, whether or not the event is related to the study or the investigational product/procedure, or is expected;
(b) a serious adverse event ("**SAE**"), which is an AE that: (i) results in death; (ii) is life-threatening; (iii) requires inpatient hospitalization or prolongation of existing hospitalization; (iv) results in persistent or significant disability or incapacity; (v) results in a congenital anomaly or birth defect; or (vi) in the professional medical judgment of an investigator, may seriously jeopardize a subject's health or may

require medical intervention to prevent any of the events listed in (i) to (v) above; or

(c) a suspected unexpected serious adverse reaction ("**SUSAR**"), which is a SAE that is unexpected according to the available information and is suspected to be causally related to an investigational product/procedure.

9.6.3 Reporting of SAEs at Investigator's Study Site: The IRB/REC has the responsibility to protect subjects' safety through review of SAEs occurred on subjects recruited at the study sites under its jurisdiction. An investigator shall, during the period of a study, have the responsibility to report to the IRB/REC all SAEs observed from any subject recruited from his/her study site in accordance with the requirements set out in Appendix 9 and process set out in Appendix 10 by using the IRB/REC's specified form.

9.6.4 Follow-up of SAEs: The investigator shall, with respect to each SAE occurred at his/her study site and reported to the IRB/REC, have the responsibility to:

(a) provide further information about the SAE on the IRB/REC's request; and
(b) follow the SAE until resolution or conclusion of the event, and provide follow-up report(s) to the IRB/REC by fifteen (15) working days after initial SAE reporting.

9.6.5 Review Schedule of SAEs: A review of the SAE report shall be performed by reviewers in the SAE Panel upon receipt of a SAE Report submitted by the investigator.

9.6.6 Assignment of Reviewer(s) for SAEs: For each SAE report assigned for SAE Panel review, the Secretariat will send the SAE report (together with all the relevant documents) to two (2) reviewers in the SAE Panel for review.

9.6.7 Scope of Considerations for SAEs: In performing a SAE report review, the reviewers will evaluate each SAE report representing the interest of subjects and not that of the hospital/institution for the purpose of protecting the rights, safety and well-being of human subjects and shall judge each SAE report to determine if investigator should report the SAE to the Hospital Authority Advanced Incident Reporting System (HA AIRS).

9.6.8 Resolution of SAE Queries: In the event that a request for clarification or further information is made by the SAE Panel reviewer(s), the Secretariat will, within ten (10) working days from the date of the request, send the request to the principal investigator. The principal investigator is required to feedback on the request in

writing as soon as possible. The request will be deemed satisfactorily addressed by the principal investigator if no further comment/query is made by the reviewer(s) within a reasonable period as determined by the Secretariat. In the event that the principal investigator does not feedback on the request within fourteen (14) days from the date of the request, the review may be terminated by the IRB/REC. In the event that the request is not deemed by all the reviewer(s) satisfactorily addressed by the principal investigator, further queries may be made to the principal investigator, or the SAE report may be channeled for full review.

9.6.9 Notification of SAE Reports: An acknowledgement of receipt of the submission of a SAE report will be issued to the principal investigator by email from the Chairman or a Vice/Deputy Chairman (or designee) as soon as possible and within ten (10) working days after the submission is made. In cases where there is no further concern or comment on a SAE report, a letter will be issued to the principal investigator.

9.6.10 Documentation of SAE Report Review: The Secretariat will be responsible for documenting and maintaining records for review of each SAE report, such as:

(a) the list of reviewers;
(b) each reviewer's conflicts of interest declarations; and
(c) the notification in writing (e.g. email of acknowledgement).

9.6.11 Reporting of SUSARs outside Investigator's Study Sites: The IRB/REC also has the responsibility to protect subjects' safety through review of SUSARs occurred outside study sites under its jurisdiction. A principal investigator shall, during the period of a study, have the responsibility to report to the IRB/REC all SUSARs reported from outside the principal investigator's study site in accordance with the requirements set out in Appendix 9.

9.6.12 Review of SUSARs: A SUSAR report will be reviewed by reviewer(s) in the SAE Panel. Review process shall be performed by two (2) reviewers in the SAE Panel upon receipt of a SUSAR report submitted by the principal investigator. In the event that the reviewer(s) deem(s) a safety report has any significant implication on protection of subjects' safety, the report will be channeled for full review. In no circumstance a study can be terminated only by expedited review.

9.6.13 Notification of SUSARs Decisions: The decision on a SUSAR submission will be notified to the principal investigator by the Chairman or a Vice/Deputy Chairman (or designee) in writing as soon as possible and within ten (10) working days after the decision is made. In case there is no any concern or comment on a SUSAR report, an

acknowledgement of receipt of the submission will be issued to the principal investigator.

9.7 Final Review

9.7.1 Final Report: The IRB/REC shall have the responsibility to follow each approved clinical study until its completion or early termination. A principal investigator shall have the responsibility to submit a final report on his/her study to the IRB/REC within two (2) months from the date of formal closure of the study by using the IRB/REC's specified form. The final report shall include a summary of study information, such as:

(a) the status of the study (e.g. completed or prematurely terminated);
(b) the numbers of subjects recruited in, withdrew from and completed the study;
(c) summary of serious adverse events;
(d) summary of complaints by subjects; and
(e) summary of significant updated information that may affect the safety of subjects.

9.7.2 Review of Final Report: Each final report will be reviewed by reviewer(s) in the Expedited Panel through an expedited review process as stipulated in Section 8.5. In the event that the reviewer(s) deem(s) any information in a final report may be linked with a substantially higher degree of risk and a full review is required, the submission will be channeled for full review.

9.7.3 Notification of Decisions: The decision on a submission will be notified to the principal investigator by the Chairman or a Vice/Deputy Chairman (or designee) in writing as soon as possible and within ten (10) working days after the decision is made. In case there is no any concern or comment on a final report, an acknowledgement of receipt of the submission will be issued to the principal investigator.

9.7.4 Failure to Submit Final Report: In the event that a principal investigator fails to submit a final report to the IRB/REC by the deadline, the IRB/REC may:

(a) refuse accepting any new application for initial review of clinical study submitted by the principal investigator and his/her participation in any new clinical study (whether as principal investigator, co-investigator/sub-investigator or otherwise); and/or
(b) notify the issue to the other research ethics committees under the HA, which may refuse his/her participation in any new clinical study (whether as principal

investigator, co-investigator/sub-investigator or otherwise);

until the final report is properly submitted and an acknowledgement is received from the IRB/REC.

10. Study Site Auditing

10.1 Purpose and Types of Audits by IRB/REC

10.1.1 Purpose of Audits by IRB: An audit by the IRB/REC is a systematic and independent examination of clinical study activities, documents and facilities to determine whether the study concerned was conducted according to its study protocol, the Declaration of Helsinki, the ICH GCP (if applicable) and the IRB/REC's requirements, for the ultimate purpose of protecting the rights, safety and well-being of the subjects participated or participating in the study.

10.1.2 Types of Audits: The IRB/REC may perform two types of audits, including:

(a) routine audits; and
(b) for-cause audits.

10.1.3 Routine Audits: Routine audits may be performed as a general quality control measure for ensuring compliance in the conduct of a clinical study at a study site. The IRB/REC will select studies for routine audits by a risk-based approach by considering various risk factors. Examples include:

(a) studies involving special ethical concerns;
(b) studies involving special clinical risk; and
(c) studies involving a large number of subjects.

10.1.4 For-cause Audits: The IRB/REC may perform a for-cause audit in response to a particular compliance concern that may be triggered by:

(a) a complaint by a subject (or his/her family member or legally acceptable representative); or
(b) a report from the study's sponsor or a competent regulatory authority in respect of any compliance concern.

10.2 Conduct and Follow-up of Audits

10.2.1 Responsibility to Facilitate Audits: Principal investigators shall allow and facilitate audits by the IRB/REC on reasonable request. An audit will be performed by auditor(s) delegated by the IRB/REC.

10.2.2 Preparation for Audits: To prepare for an audit, the IRB/REC will:

(a) liaise with the principal investigator on the scope, schedule and arrangements for the audit; and
(b) inform the principal investigator of the documents, records, materials and facilities that need to be made available to the auditor(s) during the audit.

10.2.3 Facilitation of Audits: During an audit, the principal investigator will be required to:

(a) participate in (or authorize a designee to participate in) the audit; and
(b) cooperate with the auditor(s) to facilitate a smooth audit.

10.2.4 Follow-up on Audits: After completion of an audit, the IRB/REC will issue a written audit report to the principal investigator. The principal investigator will be required to:

(a) respond on any issue or finding highlighted in the audit report;
(b) take proper follow-up action(s) with respect to each issue or finding; and
(c) issue a follow-up report to the IRB/REC upon completion of all follow-up action(s).

11. Reevaluation Mechanism

11.1 Right to Request for Reevaluation

11.1.1 Fair and Independent Review and Oversight: The IRB/REC is authorized by the Governing Body(ies) to perform ethics and scientific review and oversight of clinical studies, and will use its best endeavors to perform review and oversight in a fair and independent manner in accordance with the standards and requirements set out in this SOP.

11.1.2 Investigators' Right to Request for Reevaluation: In the event that a principal investigator does not agree with the IRB/REC's decision(s)/opinion(s) with respect to his/her clinical study (e.g. disapproval of an initial application for a study), the principal investigator will have the right to make a written request for reevaluation within thirty (30) days from the date of the IRB/REC's written notification of its decision(s)/opinion(s), provided that sufficient justification(s) for the request can be

made available to the IRB/REC for reevaluation.

11.2 Reevaluation Process

11.2.1 Initiation of Reevaluation: Any request for reevaluation shall be made in writing to the Chairman through the Secretariat. The principal investigator shall provide sufficient justification(s) for the request, with supporting documents or information as appropriate.

11.2.2 Reevaluation and Decisions: The IRB/REC will perform an independent review of the case by full review in accordance with the standards and requirements set out in this SOP, and will duly consider the rationale of the decision(s)/opinion(s) in the initial review and the justification for reevaluation by the principal investigator. The IRB/REC's decision after the reevaluation shall be final.

12. Review Fees

12.1 Determination of Review Fees

12.1.1 Determination of Review Fees: The fees for receipt of applications/submissions and performance of ethics and scientific review and oversight shall be determined and may be adjusted from time to time by the RC and CMC.

12.1.2 Notification of Review Fees: The Secretariat will have the responsibility to maintain an updated fees schedule and provide the updated information to investigators on their request.

12.2 Payment of Review Fees

12.2.1 Payment Methods: All review fees shall be paid according to the instructions of the Secretariat.

12.2.2 No Refund: No refund of any fee paid to the IRB/REC will be given in any circumstances, irrespective of the decisions/opinions of the IRB/REC, withdrawal of applications/submissions by principal investigators, refusal of applications/submissions by the IRB/REC or otherwise.

13. Records Management

13.1 Central Electronic Database

13.1.1 Central Database: A central electronic database for the clinical studies reviewed by

the IRB/REC was established and is being maintained by the IRB/REC. The database contains basic information about reviewed clinical studies (whether approved, disapproved, ongoing or closed), such as:

(a) IRB/REC reference numbers;
(b) study identifiers (e.g. study protocol titles/numbers);
(c) names and affiliated institutions of principal investigators;
(d) dates of initial review;
(e) dates of approval/decision; and
(f) dates of study closure.

13.1.2 Maintenance of Database: The Secretariat is responsible for maintaining an updated central electronic database and making the data available to the RC and CMC and the Governing Body(ies) as required.

13.2 Records Retention

13.2.1 Retention of Essential Records: The IRB/REC shall retain all essential documents and records relating to ethics and scientific review and oversight of each clinical study, including:

(a) documents and records relating to initial review of the study (e.g. initial application, study documents submitted by the principal investigators, review meeting minutes, list of reviewers and their conflicts of interest declaration, relevant correspondences between the IRB/REC and principal investigator, and the IRB/REC's written decision(s)/opinion(s));
(b) documents and records relating to continuous oversight of the study (e.g. records for review of amendments/changes, new information or deviations/compliance incidents, SUSAR reports, progress reports and final report); and
(c) documents and records of study audits by the IRB/REC (e.g. audit reports and records of follow-up actions), if applicable.

13.2.2 Records Retention Period for Approved Studies: All essential IRB/REC records with respect to each approved clinical study shall be retained for a minimum period of three (3) years from the earlier of:

(a) the date of the final report to the IRB/REC; or
(b) the date of termination of the study by the IRB/REC.

13.2.3 Records Retention Period for Disapproved Studies: All essential IRB/REC records

with respect to each disapproved clinical study shall be retained until the earlier of:

(a) the expiry of the 30-day period after the written notification of the IRB/REC’s decision(s)/opinion(s) (to allow the principal investigator to make a request for reevaluation as per Section 11.1); and
(b) the conclusion of a reevaluation as per Section 11.2.

Appendices

Blank Page

Appendix 1:
List of Defined Terms

21 CFR 56	Code of Federal Regulations Title 21 Part 56, U.S.
45 CFR 46	Code of Federal Regulations Title 45 Part 46, U.S.
AE	Adverse event
Belmont Report	Ethical Principles and Guidelines for the Protection of Human Subjects of Research officially created by the former U.S. Department of Health, Education, and Welfare
CFDA	China Food and Drug Administration
CHAIR	Consortium on Harmonization of Institutional Requirements for Clinical Research
CHAIR Phase 1 Guideline	Guideline on Ethics Oversight and Scientific Evaluation of Phase 1 Clinical Trials issued by CHAIR
Chairman	Chairman of the IRB/REC
China GCP	China Good Clinical Practice Guideline for Drug Clinical Trials
Declaration of Helsinki	Declaration of Helsinki of the World Medical Association
DSMC	Data and safety monitoring committee of a clinical study
Expedited Panel	Expedited Review Panel of the IRB/REC
RC and CMC	Research Committee (CUHK) and Cluster Management Committee (NTEC) overseeing the IRB/REC
Governing Body(ies)	The governing body(ies) of the IRB/REC, which refers to CUHK and NTEC in this SOP
HA	Hospital Authority
HA Guide	Hospital Authority Guide on Research Ethics for Study Site & Research Ethics Committee
NTEC	New Territorries East Cluster
HAHO	Hospital Authority Head Office
HAREC	Hospital Authority Research Ethics Committee
CUHK	The Chinese University of Hong Kong
The Joint CUHK-NTEC CREC	The Joint Chinese University of Hong Kong - New Territores East Cluster Clinical Research Ethics Committee
ICH GCP	International Conference on Harmonization of Technical Requirements for the Registration of Pharmaceuticals for Human Use Guideline for Good Clinical Practice
IMP	Investigational medicinal product
Independent Member	Independent member of the IRB/REC
IRB/REC	Institutional Review Board or Research Ethics Committee established by the Governing Body(ies), which refers to The Joint CUHK-NTEC CREC in this SOP
JSC	Joint Scientific Committee for Phase 1 Clinical Trials
NIH	National Institutes of Health, U.S.
Non-Scientific Member	Non-scientific member of the IRB/REC
OHRP	Office for Human Research Protections, U.S.
Phase 1 Panel	Phase 1 Clinical Trials Review Panel of the IRB/REC
SAE	Serious adverse event
SAE Panel	SAE Review Panel of the IRB/REC
Scientific Member	Scientific member of the IRB/REC
Secretariat	Secretariat of the IRB/REC

SOP	Standard operating procedure
SRP	Scientific Review Panel formed under the JSC
Standard Panel	Standard Review Panel of the IRB/REC
SUSAR	Suspected unexpected serious adverse reaction
Vice/Deputy Chairman	Vice or Deputy Chairman of the IRB/REC

Appendix 2:
Major Premises Covered under this SOP

The premises covered under this SOP shall include (but not limited to):

Institution	Address
The Chinese University of Hong Kong	The Chinese University of Hong Kong, Shatin, NT, Hong Kong
**Hospitals**	**Address**
Alice Ho Miu Ling Nethersole Hospital	11 Chuen On Road, Tai Po, NT, Hong Kong
Bradury Hospice	17 A Kung Kok Shan Road, Shatin, NT, Hong Kong
North District Hospital	9 Po Kin Road, Sheung Shui, NT, Hong Kong
Prince of Wales Hospital	30-32 Ngan Shing Street, Shatin, NT, Hong Kong
Shatin Hospital	33 A Kung Kok Street, Ma On Shan, Shatin, NT, Hong Kong
Cheshire Home	30 A Kung Kok Street, Ma On Shan, Shatin, NT, Hong Kong
Tai Po Hospital	9 Chuen On Road, Tai Po, NT, Hong Kong
**Clinics**	**Address**
Fanling Family Medicine Centre	1/F, Fanling Health Centre, 2 Pik Fung Road, Fanling, New Territories
Lek Yuen General Out-patient Clinic	G/F, 9 Lek Yuen Street, Shatin
Ma On Shan Family Medicine Centre	G/F, 609 Sai Sha Road, Ma On Shan
Wong Siu Ching Family Medicine Centre	G/F, 1 Po Wu Lane, Tai Po

Appendix 3:
Organization Chart of The Joint CUHK-NTEC CREC

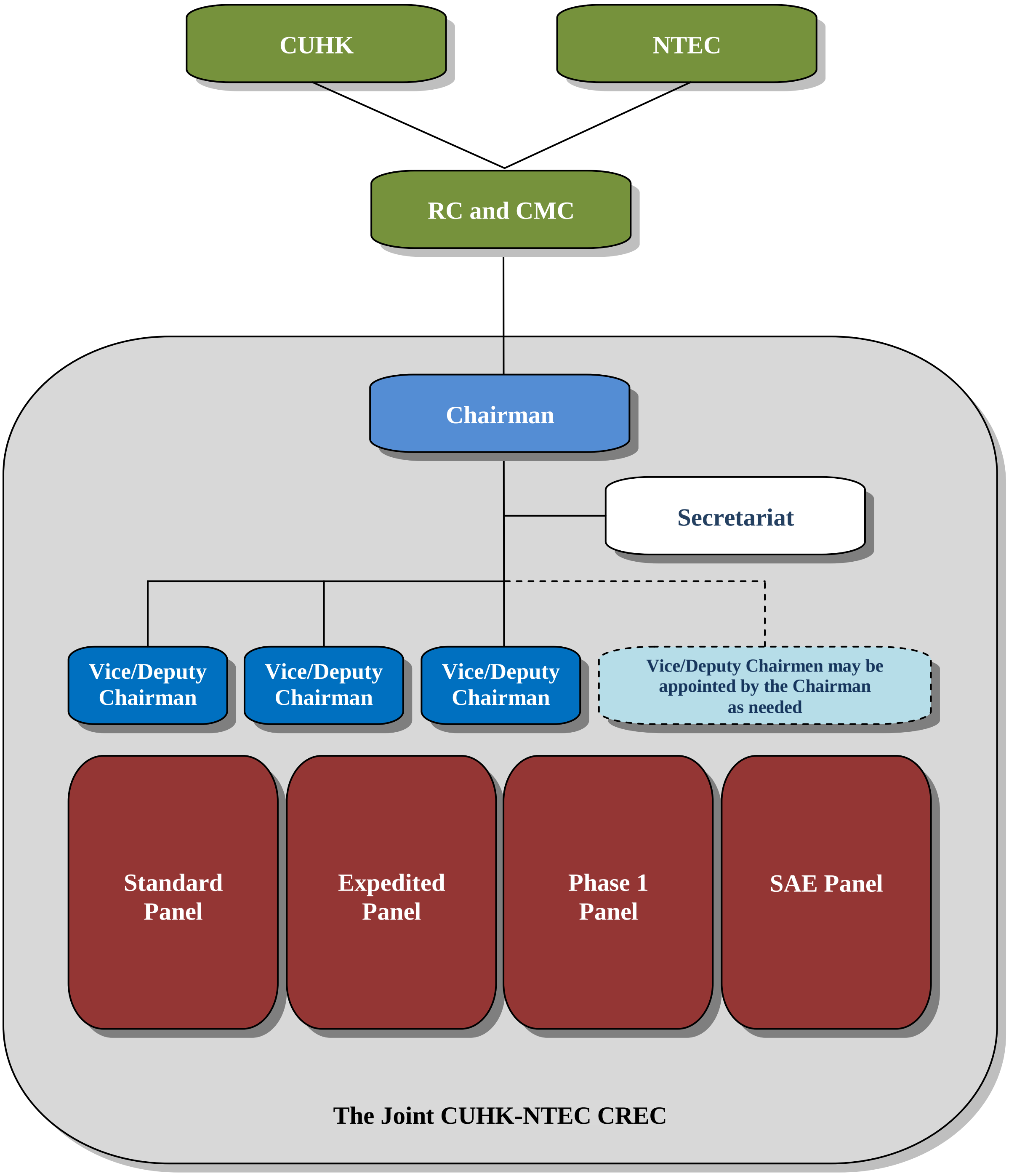

RC and CMC	Research Committee (CUHK) and Cluster Management Committee (NTEC)
NTEC	New Territorries East Cluster
CUHK	The Chinese University of Hong Kong
The Joint CUHK-NTEC CREC	The Joint Chinese University of Hong Kong - New Territores East Cluster Clinical Research Ethics Committee

Appendix 4:
Persons Eligible to Nominate IRB/REC Members

The Joint CUHK-NTEC CREC
• Chairman, The Joint CUHK-NTEC CREC
**CUHK**
• Dean, Faculty Medicine, CUHK
**NTEC**
• Cluster Chief Executive, NTEC

Appendix 5:
Documents Required for an Application for Initial Review

Documents *(See notes overleaf)*	Languages		Formats	
	English	Chinese	Hard Copy	Soft Copy
1. Submission letter for initial review	✓		✓	✓
2. Clinical research ethics review application form	✓		✓	✓
3. Crossed cheque/bank draft for payment of initial review application fee			✶	
4. Investigator's conflicts of interest declaration form	✓		✓	
5. Principal Investigator and Co-Investigator(s) curriculum vitae	✓		✓	
6. Clinical study categorization form	✓		✓	
7. Clinical study protocol	✓		✓	
8. Investigator's brochure	✶		✶	
9. Informed consent form and/or subject information sheet	✶	✶	✶	
10. Subject recruitment materials (e.g. subject recruitment advertisement or poster)	✶	✶	✶	
11. Documents/materials for use by subjects in the study (e.g. subject-administered questionnaire or diary(ies))	✶	✶	✶	
12. Certificate of insurance for clinical study	✶		✶	
13. Indemnity agreement	✶		✶	

✓ = mandatory ✶ = required if applicable

Remarks on the Documents Required for an Application for Initial Review

Documents	Remarks
1	A sample is downloadable from http://www.crec.cuhk.edu.hk.
2	The form is downloadable from http://www.crec.cuhk.edu.hk.
3	Application fee is only applicable to industry-sponsored clinical studies. Any crossed cheque or bank draft issued shall be denominated in Hong Kong dollars or U.S. dollars.
4	All investigators participating in a clinical study shall provide their signed conflicts of interest declaration forms (downloadable from http://www.crec.cuhk.edu.hk). An investigator’s potential conflicts of interest in a clinical study may include (i) any proprietary interest in the study and/or the investigational product(s)/procedure(s) (e.g. patent); (ii) any equity interest in an organization owning the rights to the study and/or the investigational product(s)/procedure(s) (e.g. stocks and options) , except for indirect ownership through collective investment schemes (e.g. mutual funds and mandatory provident funds) in which the investigator has no control over the investment strategy; (iii) any financial payment or valuable provided by an organization owning the rights to the study and/or the investigational product(s)/procedure(s) other than the costs for running a clinical study (e.g. donation); (iv) any financial arrangement linking to the outcomes of a clinical study (e.g. royalty fee); and (v) any decision-making or influential position in an organization owning the rights to the study and/or the investigational product(s)/procedure(s); and (vi) a direct family relationship with a person having any of the above interests (e.g. spouse).
5	The principal investigator and co-investigator’s updated curriculum vitae must be submitted.
6	The form is downloadable from http://www.crec.cuhk.edu.hk.
7, 8	Incorporation of an investigator brochure with a clinical study protocol is acceptable.
9	Informed consent form and subject information sheet may be combined into one document. A checklist is downloadable from http://www.crec.cuhk.edu.hk.
10, 11	The language(s) used in any subject recruitment material and/or other document/material for use by subjects will depend on the language(s) of the target subject population.
12	Any clinical study with higher than nominal clinical risk as determined by the IRB/REC may be required to be covered by appropriate insurance policy(ies) (e.g. no-fault clinical trial insurance), evidenced by certificate(s) of insurance.<br>A certificate of insurance may be submitted to the IRB/REC separately from the application subject to the IRB/REC’s permission, but in any event shall be prior to initiation of the clinical study.
13	An indemnity must be provided by the sponsor of an industry-sponsored clinical study according to the mandatory requirement of the HA. The standard indemnity agreements are downloadable from http://www.crec.cuhk.edu.hk.<br>A fully executed indemnity agreement may be submitted to the IRB/REC separately from the application subject to the IRB/REC’s permission, but in any event shall be prior to commencement of the clinical study.

Appendix 6:
Clinical Study Categorization Form

The Joint Chinese University of Hong Kong - New Territores East Cluster Clinical Research Ethics Committee

Clinical Study Categorization Form

Risk Group	Risk Factors (See notes overleaf)		Yes	No
Human Subjects	1	Recruitment of human subjects [***see notes of completion***]	☐ →2	☐ →B
Medical Products	2	Use of any medical product that is not needed or used for the Subjects' normal clinical care [***see notes of completion***]	☐ →3	☐ →8
	3	Each medical product used is registered or permitted to be marketed in Hong Kong	☐ →4	☐ →5
	4	Use of each medical product is within the labeled use in Hong Kong [***see notes of completion***]	☐ →8	☐ →5
	5	Any medical product used is a chemical or biological drug that is to be tested in humans for the first time	☐ →C	☐ →6
Study Designs	6	The study is a phase 1 clinical trial on a chemical or biological drug as designated on its study protocol	☐ →C	☐ →7
	7	The study only has human pharmacology, tolerability and/or safety (but not efficacy) of the chemical or biological drug as its primary objective(s) as specified on its study protocol	☐ →C	☐ →8
	8	Involvement of placebo, impeding access to available treatment, or withdrawal of ongoing treatment driven by the study protocol	☐ →A	☐ →9
Clinical Procedures	9	Involvement of any clinical procedure that is not needed or applied for the subjects' normal clinical care [***see notes of completion***]	☐ →10	☐ →11
	10	Each clinical procedure applied presents no more than minimal clinical risk to the subjects [***see notes of completion***]	☐ →11	☐ →A
Subject Assignment Methods	11	Subjects are assigned to different clinical methods/strategies (i.e. therapies, prophylaxes or diagnoses) by randomization or other research specific methods (other than by the professional judgment of qualified medical professionals)	☐ →A	☐ →12
Subject Vulnerability	12	Involvement of vulnerable subjects [***see notes of completion***]	☐ →A	☐ →B

**Channel A**	**Full review by Standard Panel** (unless otherwise determined by the IRB/REC according to the IRB/REC's SOP)
**Channel B**	**Expedited review by Expedited Panel** (unless otherwise determined by the IRB/REC according to the IRB/REC's SOP or requested by the principal investigator for a full review)
**Channel C**	**Full review by Phase 1 Panel** (unless otherwise determined by the IRB/REC according to the IRB/REC's SOP) / Recommend the principal investigator to collaborate with a study site under the jurisdiction of a research ethics committee with a specific review panel for phase 1 clinical trials

Official Use Only
**Categorization by IRB/REC:** ☐ **Channel A** ☐ **Channel B** ☐ **Channel C**
**Reason** (if IRB/REC applies a different categorization):

Notes for Completion of the Clinical Study Categorization Form

Risk Factors	Remarks
1	Recruitment of human subjects means prospective recruitment of subjects into a clinical study, irrespective of the nature of the study. Retrospective research on human materials or human data that have already been collected may not require recruitment of human subject unless separate informed consent is required for some or all of the subjects in the circumstances.
2	Medical products may include (but not limited to):<br>(a) drugs (e.g. chemical drugs, biological drugs and vaccines);<br>(b) medical devices (e.g. implants, diagnostic kits and imaging machines)<br>(c) Chinese/herbal medicines (e.g. proprietary/traditional Chinese medicines);<br>(d) health/nutritional supplements;<br>(e) cell therapies (e.g. stem cells); and<br>(f) gene therapies (e.g. viral vectors).
4	Labeled use refers to the use a medical product in accordance with the conditions of registration in Hong Kong (e.g. indications, patient groups, formulations and dosages).
9	Clinical procedures include (but not limited to):<br>(a) clinical examination/assessments (e.g. venipuncture)<br>(b) surgical procedures (e.g. tumor resection);<br>(c) nursing procedures;<br>(d) physiotherapies;<br>(e) occupational therapies;<br>(f) psychotherapies;<br>(g) behavioral therapies;<br>(h) alternative therapies (e.g. acupuncture); and<br>(i) imaging methods (e.g. X-ray examination).
10	Minimal clinical risk means the probability and magnitude of harm or discomfort anticipated to be caused to the human subjects are not greater than those ordinarily encountered in their daily life or normal clinical care (e.g. the clinical risk associated with a buccal swab, taking of a small quantity of blood by venipuncture, and a chest x-ray examination).
12	Vulnerable subjects are individuals whose willingness to participate in clinical studies may relatively easily be unduly influenced by biases or coercive factors, or who are incapable of giving free informed consent through a normal informed consent process, such as:<br>(a) children or adolescent (of less than 18-year-old);<br>(b) illiterates;<br>(c) mentally incapacitated persons;<br>(d) impoverished persons;<br>(e) ethnic minority groups;<br>(f) patients in emergency conditions;<br>(g) prisoners; and<br>(h) subordinates or students of investigators.

Appendix 7:
Common Considerations in IRB/REC Review

Key Dimensions	Common Considerations
Research Products/Procedures	• Involvement of clinical interventions (e.g. medical products or clinical procedures)<br>• Potential risks and related scientific rationale<br>• Potential benefits and related scientific rationale
Study Design	• Significance of research questions<br>• Correlation of study design and research questions<br>• Use of randomization or other research specific subject assignment methods<br>• Involvement of placebo, impeding access to available treatment, or withdrawal of ongoing treatment driven by study protocol<br>• Statistical considerations
Study Execution	• Expertise and experience of investigators and study personnel<br>• Training on the Declaration of Helsinki and ICH GCP (if applicable)<br>• Study site facilities<br>• Mechanism of ongoing safety monitoring and reporting<br>• Medical emergency arrangements
Subjects' Rights	• Subject type and vulnerability<br>• Involvement of healthy volunteers or subjects without the targeted diseases/conditions<br>• Subject recruitment strategies<br>• Informed consent documents and process<br>• Protection of subjects' personal data<br>• Payments to subjects
Potential Research Biases	• Conflicts of interest, potential conflicts of interest and declaration of interest<br>• Public disclosure of study information (e.g. by registration with public clinical trial registries)<br>• Publication plan
Potential Liability Management	• Insurance<br>• Indemnity (for industry-sponsored clinical studies)

Appendix 8:
Sample Notice for Communicating IRB/REC's Decisions

香港中文大學醫學院

Faculty Of Medicine

The Chinese University Of Hong Kong

醫院管理局
新界東醫院聯網
Hospital Authority
New Territories East Cluster

The Joint Chinese University of Hong Kong - New Territores East Cluster Clinical Research Ethics Committee

香港中文大學-新界東醫院聯網 臨床研究倫理 聯席委員會

8/F, Lui Che Woo Clinical Sciences Building, Prince of Wales Hospita, Shatin HK
Tel: 2632 3935 Fax: 2646 6653 Website: http://www.crec.cuhk.edu.hk

The Joint CUHK-NTEC CREC is an independent committee established by CUHK/NTEC and authorized to perform ethics and scientific review and oversight of clinical studies within the jurisdiction of CUHK/NTEC in accordance with its standard operating procedure and the principles of the Declaration of Helsinki and ICH Good Clinical Practice.

IRB/REC Ref. No.: <Ref. No> <Chop Date>

To: <PI Name>
<PI Title & Department>
<PI Affiliated Institution>

This notice is issued by The Joint CUHK-NTEC CREC with respect to the application/submission by you, being the principal investigator of the following study at your study site:

- Study Protocol Title: <Title>
- Study Protocol No.: <No.>
- Investigator(s) <PI and Co-I(s)>
- Academic Supervisor: (if applicable) <Name of Academic Supervisor, for study project only >
- Site Supervisor: (if applicable) <Name of Site Supervisor, for study project only >

In accordance with our standard operating procedure, we have duly performed ethics and scientific review of your application/submission as detailed below:

- Nature of Your Application/Submission: ☐ Initial application ☐ Amendments/changes ☐ Others: ☐ Renewal
- Mode of Review: ☐ Full review ☐ Expedited review
- Date of Initial/Renewal Approval: <Date of Decision>
- Date of Amendment: (if applicable) <Date of Decisionl>
- Document(s) Reviewed: (if applicable) <"See Schedule 1">
- Reviewer(s): <"See Schedule 2" or List>

After due review by our reviewer(s), we hereby write to inform you of our decision on your application/submission as follows:

• Decision:	☐ Application/Submission approved<br>☐ Application/Submission approved with condition(s) (see condition(s) below)<br>☐ Application/Submission approved with remark(s) (see remark(s) below)
• Condition(s):<br>(if applicable)	<State condition(s), or put "N/A" if not applicable>
• Remark(s):<br>(if applicable)	<State remark(s), or put "N/A" if not applicable>
• Regular Progress Report(s) Required:	Every <No.> months from the date of initial/renewal approval and during the period of the study if required

You, being the principal investigator of the study at your study site, are reminded to comply with our requirements and to maintain communication with us during the period of the study by undertaking the principal investigator's responsibilities including (but not limited to):

- if the study is an industry-sponsored clinical study, submitting to us a copy of the fully executed indemnity agreement satisfying the Hospital Authority's requirement prior to commencement of the study (if it has not been submitted yet);
- observing and complying with all applicable requirements under our standard operating procedure ("IRB/REC SOP"), the Declaration of Helsinki and the ICH GCP (if applicable);
- submitting regular progress report(s) at the required intervals (as specified above) in accordance with the requirements in the IRB/REC SOP;
- not implementing any amendment/change to any approved study document/material without our written approval, except where necessary to eliminate any immediate hazard to the subjects or if an amendment/change is only of an administrative or logistical nature;
- notifying us of any new information that may adversely affect the rights, safety or well-being of the subjects or the proper conduct of the study;
- reporting any deviation from the study protocol or compliance incident that has occurred during the study and may adversely affect the rights, safety or well-being of any subject in accordance with the requirements in the IRB/REC SOP;
- submitting safety reports on all SAEs observed at your study site or SUSARs reported from outside your study site in accordance with the requirements in the IRB/REC SOP; and
- submitting a final report in accordance with the requirements in the IRB/REC SOP upon completion or termination of the study at your study site.

In addition to the above, you are also reminded to observe and comply with other applicable regulatory and management requirements including (but not limited to):

- if required by Hong Kong laws or regulations, obtaining a certificate for clinical trial through the Hong Kong Department of Health and complying with the associated requirements;
- obtaining the necessary consent from the management of your institution/department in accordance with the requirements of your institution/department;
- if required by local laws or regulations at conducting site out of IRB/REC's jurisdiction, obtaining an approval and complying with associated requirements;
- not representing to any third party or in any way likely to mislead any third party forming the view that the approval from the IRB/REC has any extraterritorial effect; and

- with due diligence ensuring your teams, staff, agents or whosoever connected with you to comply with the preceding requirements.

Yours sincerely,
for and on behalf of
The Joint CUHK-NTEC CREC

____
<NAME OF CHAIRMAN/DESIGNEE>
<TITLE>

Schedule 1
Documents Reviewed

The documents reviewed by The Joint CUHK-NTEC CREC with respect to the said application/submission include:

<List documents. Include version date/no. if applicable>

Schedule 2
Reviewers List
Joint CUHK-NTEC Clinical Research Ethics Committee

Title and Name	Occupation	Qualification	Male / Female (M/F)	Study Reviewed by	Present in CREC meeting on <Date>

Appendix 9:
Safety Events Reporting Requirements

Origins of safety events:	• <u>Local Site</u>: SAEs observed from subjects of a principal investigator's own study site	• <u>Other Site(s)</u>: SUSARs reported from outside a principal investigator's own study site, (e.g. SUSARs reported from another study site in the same multicentre clinical study, or from another clinical study involving the same investigational product/procedure)
Types of safety events that need to be reported to the IRB/REC:	• All SAEs	• All SUSARs
Reporting timeline (for phase 1 clinical trials):	• Twenty-four (24) hours from the first awareness of a SAE by the principal investigator	• Thirty (30) calendar days from the date of receipt of a SUSAR report by the principal investigator
Reporting timeline (for clinical studies other than phase 1 clinical trials):	• <u>Fatal or life-threatening SAEs</u>: Twenty-four (24) hours from the first awareness of a SAE by the principal investigator<br>• <u>Other SAEs</u>: Twenty-four (24) hours from the first awareness of a SAE by the principal investigator	• Thirty (30) calendar days from the date of receipt of a SUSAR report by the principal investigator

Appendix 10:
Serious Adverse Events Reporting Process

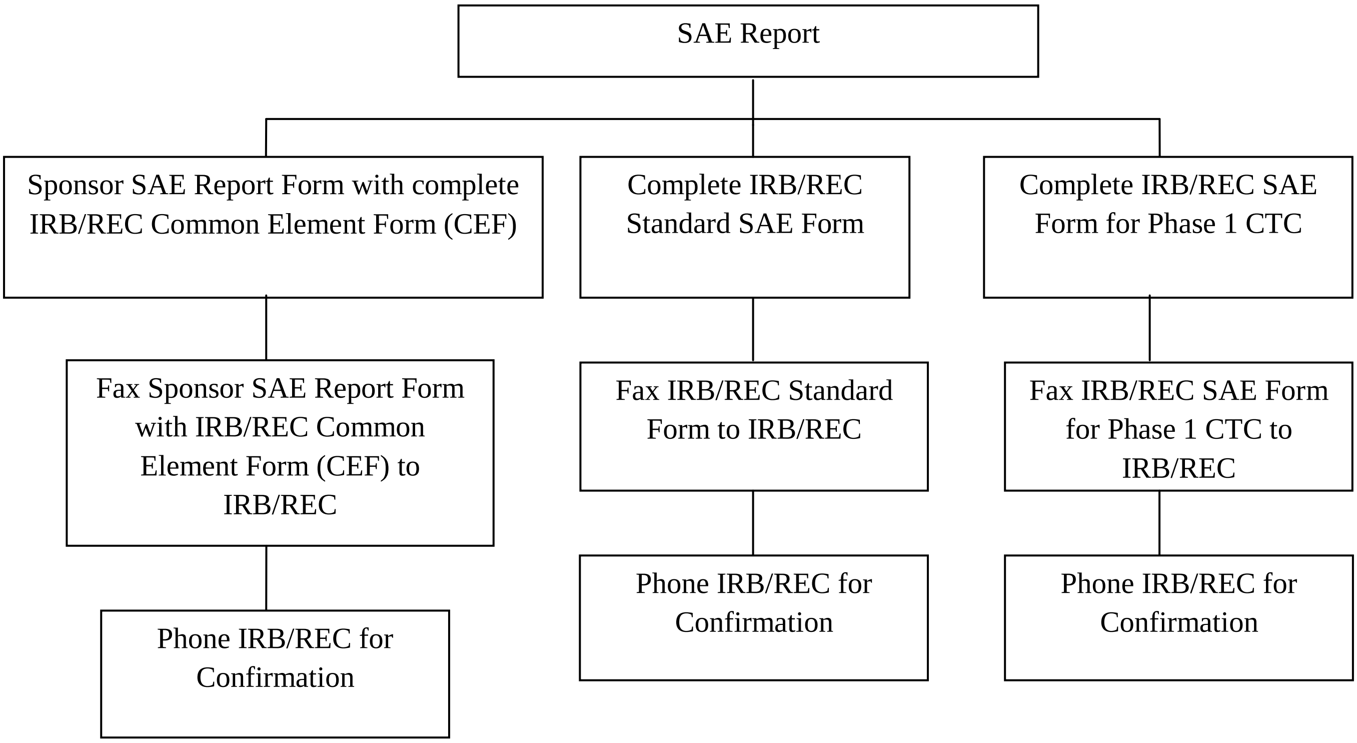

Blank Page
