# Supplementary material for: Attenuation of blood pressure in spontaneously hypertensive rats by acupuncture was associated with reduction oxidative stress and improvement from endothelial dysfunction
Source: Chin Med. 2016 Aug 30;11(1):38. doi: 10.1186/s13020-016-0110-0 (PMC5006281; doi:10.1186/s13020-016-0110-0)
Supplement: Supplementary file 5 — 10.1186/s13020-016-0110-0 The Joint CUHK-NTEC Clinical Research Ethics Committee. [file 13020_2016_110_MOESM5_ESM.docx]

**The Joint CUHK-NTEC Clinical Research Ethics Committee**

**Chairman:**

Prof. Benny C.Y. ZEE

**Vice/Deputy Chairman:**

Dr. C.K. LI

**Members:**

Prof. Alice P.S. KONG

Prof. Vivian LEE

Prof. Brigette B.Y. MA

Prof. Vincent C.T. MOK

Prof. John RUDD

Prof. Cheuk Chun SZETO

Prof. Wai Kwong TANG

Prof. Brian TOMLINSON

Prof. Eric WONG

Dr. Simon K.C. CHAN

Dr. Ernest H.M. MA

Dr. Kevin K.H. OR

Dr. Keary R. ZHOU

Ms. Emily M.L. CHAN

Mr. Bryan CHUNG

Ms. Alexandra D.W. LO

Mr. Wilson Y.L. SO

Mr. Ping Hei TAO

Mr. Foster H.C. YIM

**SAE Monitoring Members:**

Prof. Sandra S.M. CHAN

Prof. Bonnie C.H. KWAN

Prof. Cheuk Chun SZETO (Temporary)

Dr. Andrea O.Y. LUK

**Secretary:**

Ms. Jenny NG

Ms. Envy LEE
